# Metabolic Fluctuations in the Human Stool Obtained from *Blastocystis* Carriers and Non-Carriers

**DOI:** 10.3390/metabo11120883

**Published:** 2021-12-17

**Authors:** Emma L. Betts, Jamie M. Newton, Gary S. Thompson, Fakhriddin Sarzhanov, Vasana Jinatham, Moon-Ju Kim, Siam Popluechai, Funda Dogruman-Al, Eun-Jeong Won, Eleni Gentekaki, Anastasios D. Tsaousis

**Affiliations:** 1 Laboratory of Molecular and Evolutionary Parasitology, RAPID Group, School of Biosciences, University of Kent, Canterbury CT2 7NJ, Kent, UK; elb48@kent.ac.uk (E.L.B.); jmn24@kent.ac.uk (J.M.N.); G.S.Thompson@kent.ac.uk (G.S.T.); 2 Wellcome Trust Biomolecular NMR Facility, School of Biosciences, University of Kent, Canterbury CT2 7NJ, Kent, UK; 3 Faculty of Medicine, Akhmet Yassawi International Kazakh-Turkish University, Turkestan 161200, Kazakhstan; fakhriddin.sarjanov@gmail.com; 4 School of Science, Mae Fah Luang University, Chiang Rai 57100, Thailand; vasana.jinatham@gmail.com (V.J.); siam@mfu.ac.th (S.P.); 5 Department of Parasitology and Tropical Medicine, Chonnam National University Medical School, Hwasun-gun 58128, Jeollanam-do, Korea; 4_chance@naver.com (M.-J.K.); Parasite.woni@jnu.ac.kr (E.-J.W.); 6 Gut Microbiome Research Group, Mae Fah Luang University, Chiang Rai 57100, Thailand; 7 Division of Medical Parasitology, Department of Medical Microbiology, School of Medicine, Gazi University, Ankara 06490, Turkey; alfunda@yahoo.com; 8 Department of Laboratory Medicine, Chonnam National University Hwasun Hospital, Hwasun-gun, Gwangju 58128, Jeollanam-do, Korea

**Keywords:** amino acids, *Blastocystis*, inflammation, metabolomics, metabolite profiles, stool, NMR

## Abstract

*Blastocystis* is an obligate anaerobic microbial eukaryote that frequently inhabits the gastrointestinal tract. Despite this prevalence, very little is known about the extent of its genetic diversity, pathogenicity, and interaction with the rest of the microbiome and its host. Although the organism is morphologically static, it has no less than 28 genetically distinct subtypes (STs). Reports on the pathogenicity of *Blastocystis* are conflicting. The association between *Blastocystis* and intestinal bacterial communities is being increasingly explored. Nonetheless, similar investigations extending to the metabolome are non-existent.Using established NMR metabolomics protocols in 149 faecal samples from individuals from South Korea (*n* = 38), Thailand (*n* = 44) and Turkey (*n* = 69), we have provided a snapshot of the core metabolic compounds present in human stools with (*B+*) and without (*B−*) *Blastocystis*. Samples included hosts with gastrointestinal symptoms and asymptomatics. A total of nine, 62 and 98 significant metabolites were associated with *Blastocystis* carriage in the South Korean, Thai and Turkish sample sets respectively, with a number of metabolites increased in colonised groups. The metabolic profiles of *B+* and *B−* samples from all countries were distinct and grouped separately in the partial least squares-discriminant analysis (PLS-DA). Typical inflammation-related metabolites negatively associated with *Blastocystis* positive samples. This data will assist in directing future studies underlying the involvement of *Blastocystis* in physiological processes of both the gut microbiome and the host. Future studies using metabolome and microbiome data along with host physiology and immune responses information will contribute significantly towards elucidating the role of *Blastocystis* in health and disease.

## 1. Introduction

The healthy gastrointestinal (GI) tract is composed of trillions of microorganisms including bacteria, viruses, fungi and protozoa that collectively have complex roles in gut homeostasis and host health. This includes immune system modulation, protection against disease and aiding absorption and synthesis of nutrients [1,2,3,4]. When the gut ecosystem is disrupted, states of dysbiosis can ensue, which can lead to GI and systemic diseases, such as obesity, diabetes, inflammatory bowel disease (IBD) and autoimmune disease [2].

The advancement of high-throughput methodologies has led to a greater understanding of the composition of the gut microbiota [5,6], yet there is still uncertainty regarding the roles of protozoal colonizers in the gut. Historically, the presence of intestinal protozoa has been considered detrimental, and subject to elimination [7]. However, in recent years, it has been postulated that some microbial eukaryotes might modulate host immune responses and the gut environment. Moreover, some microbial eukaryotes have been associated with specific bacterial communities, which might result in disturbing gut homeostasis [8,9,10,11]. Alternatively, colonisation with certain microbial eukaryotes has been linked to a healthy gut [7,8]. One such organism is the questionable parasite *Blastocystis*, which can persist in the large intestine for prolonged periods of time. The organism has a ubiquitous distribution, and is the most commonly reported intestinal microbial eukaryote in humans, with prevalences ranging from 20% in high-income countries to over 50% in low and middle-income countries. In some cases, *Blastocystis* has been associated with GI disorders including irritable bowel syndrome (IBS) and IBD [12,13,14,15,16]. In silico and in vitro studies have revealed virulence factors suggesting a pathogenic role [17,18,19,20,21,22,23]. Nonetheless, asymptomatic colonisation in human and non-human hosts is frequently reported, where its presence has been linked to increased bacterial diversity and positive gut health [8,24,25,26,27,28]. This has resulted in its pathogenicity being disputed and instead, *Blastocystis* is increasingly considered a mutualist member of the gut microbiota [29,30,31].

While previous reports have documented the gut microbiome profiles of human hosts positively and negatively colonised with *Blastocystis*, similar investigations at the level of the metabolome are non-existent. 1D ^1^H Nuclear Magnetic Resonance (NMR) is a quantitative and reproducible metabolite detection method, which has been previously used to analyse the metabolomes of many bacterial [32], mammalian [33,34], plant [35], fungal [36] and protozoan [37,38,39] cells/organisms. In recent years, there has been a marked increase in the use of metabolomics-based studies for exploring host parasite interactions [37,40,41,42], as well as aiding research into chemotherapeutics [43,44]. NMR-based metabolomics of faecal samples provides a non-invasive, non-targeted, high-throughput approach to examine metabolite profiles of stool providing a snapshot of microbial activities.

Herein, we used ^1^H NMR metabolomics on *Blastocystis* positive (*B+*) and *Blastocystis* negative (*B−*) human stool samples to investigate metabolic profiles of *Blastocystis* colonisation for human cohorts spanning three different countries: South Korea, Thailand, and Turkey.

## 2. Results

In order to evaluate variations in metabolic profiles from *Blastocystis* carriers and non-carriers, we prepared and analysed a total of 149 faecal samples from three countries. Of the 71 samples from Turkey, 69 were successfully processed and analysed 53 *Blastocystis* positive (*B+*), 16 *Blastocystis* negative (*B−*)]; 38 (18 *B+*, 20 *B−*) from South Korea; and 43 (14 *B+*, 29 *B−*) from Thailand.

### 2.1. Metabolite Profiles

^1^H NMR metabolomics of *Blastocystis* positive (*B+*) and *Blastocystis* negative (*B−*) humans were compared against a reference database of 338 compounds from the Chenomx database. Supplementary Figure S1 shows an example of the NMR spectra from *B+* and *B−* individuals from each country. Initial analysis revealed the presence of residual ethanol in the majority of samples, indicating incomplete removal during sample preparation. Therefore, ethanol was removed from all subsequent analysis to reduce data skewing of metabolites that represent the gut environment.

Univariate analysis (Volcano plots: FDR-adj *p*-value vs. Fold change) was used to identify metabolites that varied significantly between the *B+* and *B−* sample sets within each country regardless of the health status of the individual. Significant metabolites for samples from each country are detailed below. In the Thai samples, a total of 62 metabolites were identified; of these, 55 were significantly increased and 7 were significantly decreased in *B+* samples. In the Turkish samples, a total of 98 metabolites were identified, of which 81 were significantly increased and 17 significantly decreased in *B+*. Finally, the South Korean samples had a total of nine significant metabolites, with eight significantly increased in *B+* and one that was significantly decreased.

A total of 30 metabolites (padj < 0.05) were commonly identified across two or more countries (Supplementary Table S2), while the metabolites dimethylamine and glycerol were identified in samples from all three countries. Of the metabolites identified across two or more countries, 5 (16.7%) had antithetical levels. This included 3-methylglutarate, 3-methylxanthine, acetamide, creatinine and trimethylamine N-oxide. All but trimethylamine N-oxide were significantly increased in *B+* samples from Turkey, but the same metabolites were decreased in *B+* samples from Thailand. Trimethylamine N-oxide was significantly increased in *B+* samples from Thailand but decreased in *B+* samples from Turkey. The remaining metabolites exhibited the same trend between *B+* and *B−* from different countries.

Univariate analysis was also carried out between countries. All *B+* samples were compared against all *B−* samples (*B+* vs. *B−*), regardless of symptomology (this included diarrheic *B−* samples from South Korea, which had varied diagnosed pathologies) or country. A total of 98 metabolites were identified, 29 of these were significantly increased in *B+* samples, and 69 were significantly decreased (Supplementary Table S3). Secondly, asymptomatic (no history of GI disease, no GI-related symptoms), *Blastocystis* positive (*D−B+*) samples were compared against symptomatic (diarrhetic), *Blastocystis* negative (*D+B−*) (*D−B+* vs. *D+B−*). Here, 166 significant metabolites were identified; 163 were increased in *D+B−*, while three were significantly decreased in the same group (Supplementary Table S4).

Samples from asymptomatic hosts with (*D−B+*) and without (*D−B−*) *Blastocystis* (*D−B+* vs. *D−B−*) were also compared. A total of six significant metabolites were identified, all of which were significantly increased in *B+* samples (Supplementary Table S5).

Furthermore, univariate analysis was also carried out on all *B+* samples compared against all *B−* samples. For this analysis, the *B−* samples from South Korea were excluded due to the hosts suffering from various diagnosed pathologies (other than GI-related diseases), which might affect the metabolic profiles. The remaining samples comprised *B+* (*D+* and *D−*) samples compared against *B−* (*D+* and *D−* excluding Korea *D+*). Within this sample set, a total of 51 metabolites were identified, 31 were significantly increased in the *B+* sample set, while 20 were significantly decreased (Supplementary Table S6).

**Figure 1:**
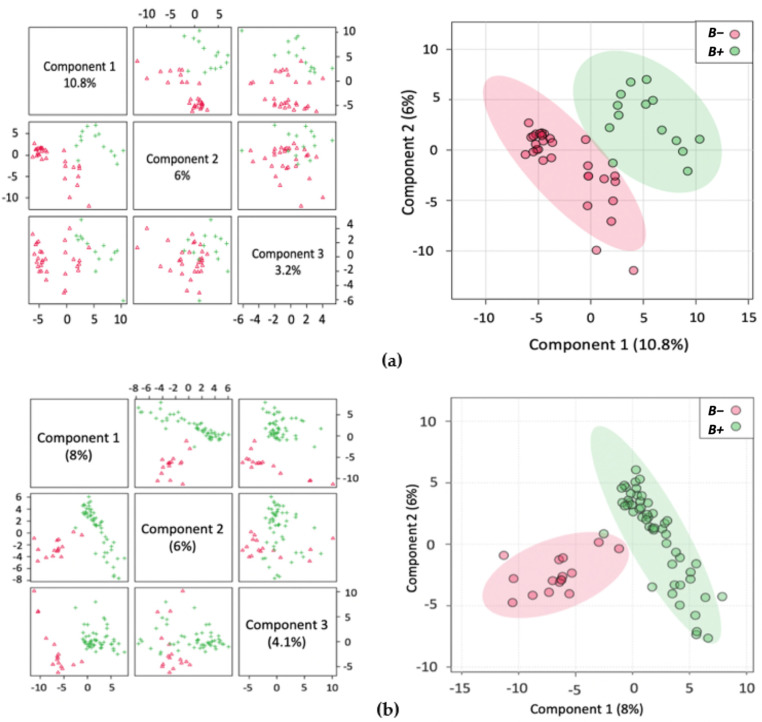

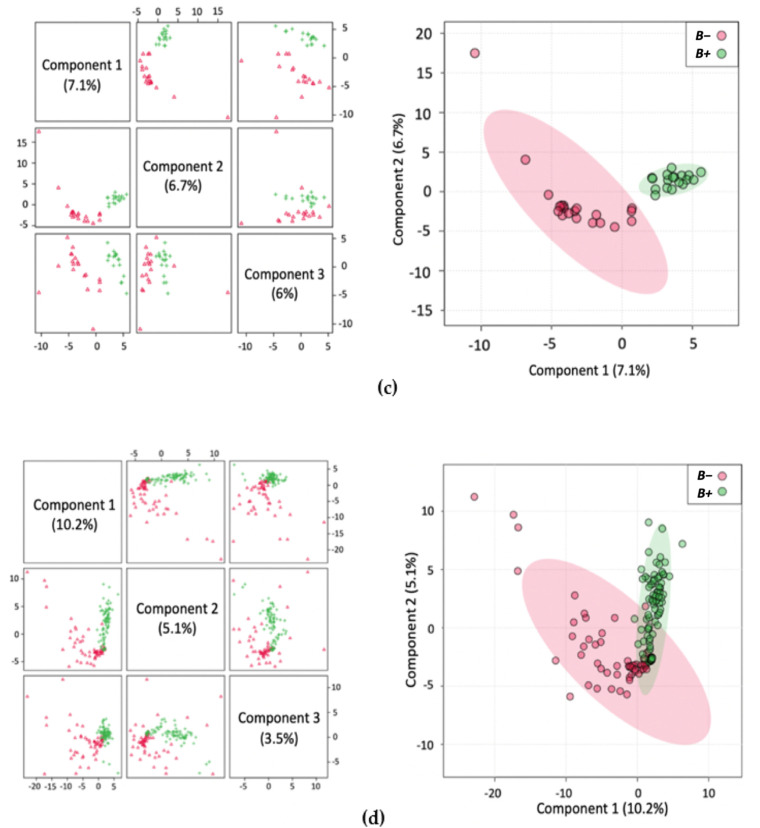
Multivariate analysis showing Partial Least Squares Discriminant Analysis (PLS-DA) with accompanying 2Dscores plots of the normalised metabolite concentrations (**a**) Thailand; (**b**) Turkey; (**c**) South Korea; (**d**) *B+* vs. *B*−.

Unsupervised principal component analysis (PCA) (Supplementary Figure S2) of the normalised metabolite concentrations was carried out on samples from each country individually as well as on the other groupings (Table 1). The variance between the groups was further investigated by way of supervised analysis using partial least squares discriminant analysis (PLS-DA) to maximize the covariance between data and group (Figure 1, Supplementary Figure S3). Results were cross-validated using Leave-One-Out Cross-Validation (LOOCV) and met R2, Q2 and Prediction Accuracy performance measure thresholds determined in Metaboanalyst (with a minimum of a two-component model).

**Figure 2:**
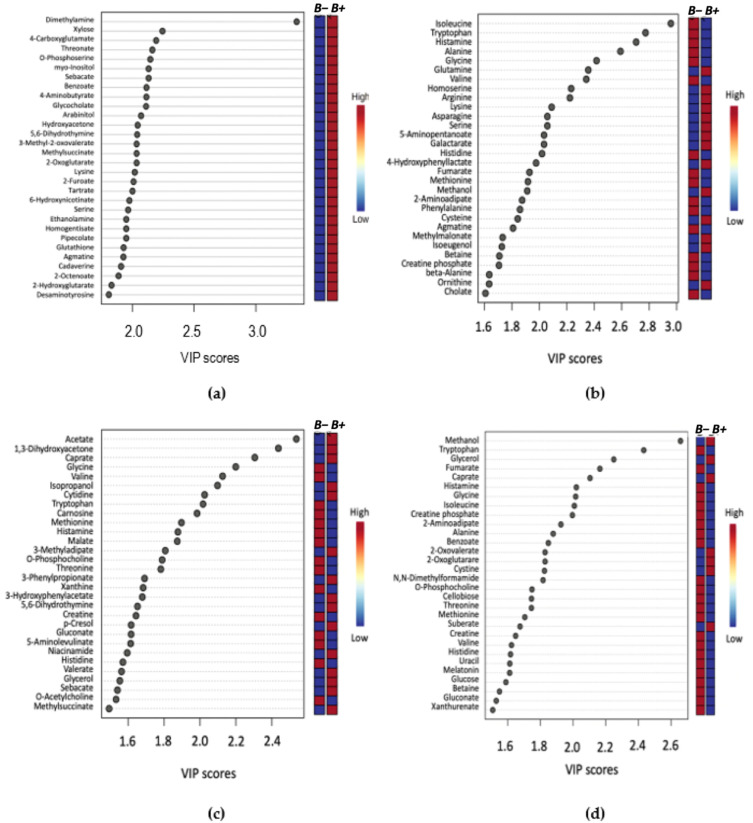
VIP score plot summarising the 30 most important metabolites determined by the PLS-DA plot. The *x*-axis indicates the VIP score corresponding to significant metabolites on the *y*-axis. The coloured boxes on the right represent the relative concentration of the metabolite for *B+* (infected) or *B−* (control) samples. (**a**) Thailand, (**b**) Turkey, (**c**) South Korea, (**d**) *B+* vs. *B*−.

The identified metabolites were further ranked by the PLS-DA Variable Importance in Projection (VIP) score (Figure 2, Supplementary Figure S4). Here, up to and including the top 30 ranked significant metabolites were identified and their relative concentrations in *B+* and *B−* samples indicated. Uniquely among all the VIP analyses, the top 30 metabolites were decreased in the *B−* and increased in the *B+* Thai samples (Figure 2a).

### 2.2. Metabolic Profile and Pathway Analysis of Blastocystis Infected Individuals

**Figure 3:**
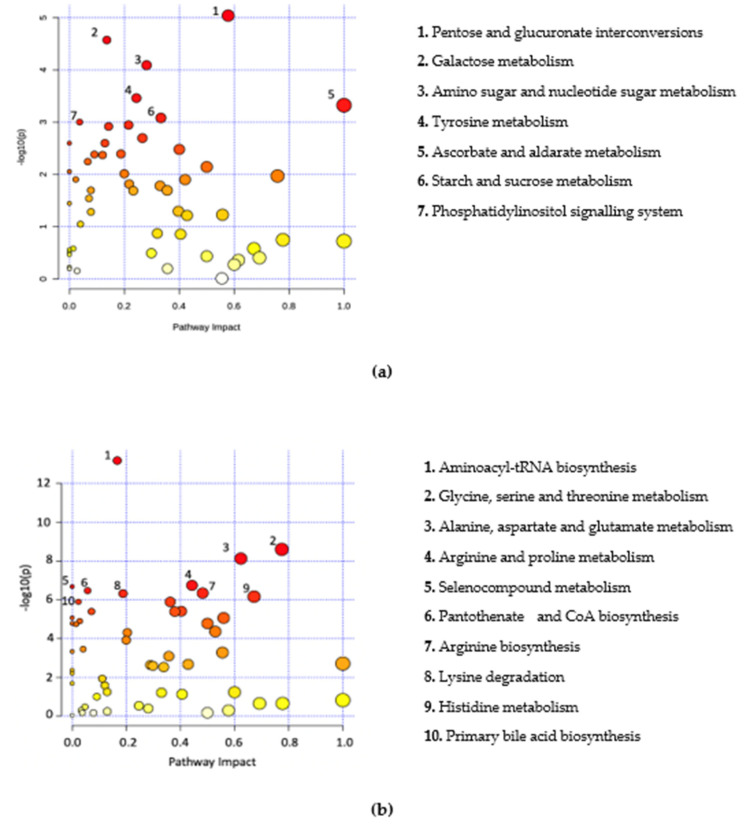

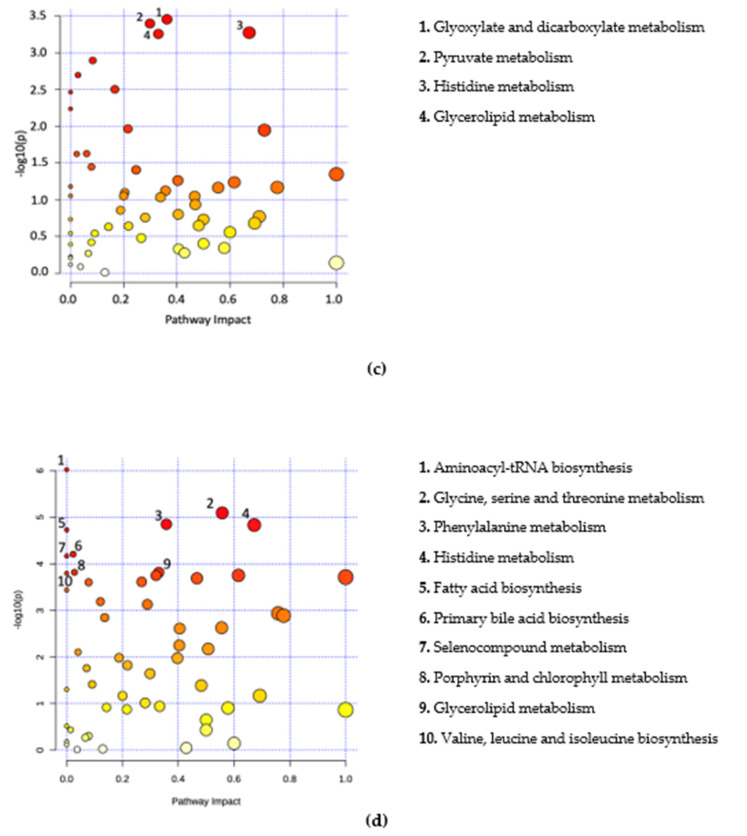
Metabolome view of pathway analysis based on pathways that involve significant metabolites. Pathway and library information was supplied from KEGG based on Homo sapiens with pathway information up to date from October 2019. The colour and size of circles are a representation of the adjusted *p*-value (colour) and the pathway impact value (circle size). Darker coloured circles (Red) represent more statistically significant findings and the transition to yellow follows the decline in significance. Larger circles have an increased pathway impact. Metabolites in the top right-hand corner are significantly changed and are likely to have an impact on the pathway. Labelled pathways include the pathways with a *p*-adjusted < 0.05 and highest pathway impact values. (**a**) Thailand, (**b**) Turkey, (**c**) South Korea, (**d**) *B+* vs. *B*−.

Significant metabolites were further analysed by way of pathway analysis. This was performed to identify possible pathways that were significantly altered in *B+* and *B−* data and were based on adjusted *p*-values from pathway enrichment analysis procedures. A total of seven metabolic pathways for Thailand, 26 pathways for Turkey, four pathways for South Korea and 22 pathways for *B+* vs. *B−* samples were identified (padj < 0.05) (Figure 3, Supplementary Tables S7–S10). All pathways were incomplete. A total of three pathways were commonly identified across two or more countries. These include glyoxylate and dicarboxylate metabolism, histidine metabolism and tyrosine metabolism. Sixteen of the pathways identified in the *B+* vs. *B−* grouping were also identified in the individual country groups.

## 3. Discussion

To our knowledge, this is the first report of a metabolomics-based study carried out on *Blastocystis* in humans. The focus was to assess metabolite profiles from *Blastocystis* carriers (*B+*) and non-carriers (*B−*) from three different countries and explore possible associations of the organism with the host stool metabolome by using ^1^H NMR spectroscopy [34,39]. Findings revealed a distinct variation in the metabolome of positive carriers compared to non-carriers, with a number of distinguishable metabolites found between the groups. Metabolites were investigated individually and also mapped to metabolic pathways, several of which were linked to *Blastocystis* presence in the gut.

A total of 62, 98 and nine significant metabolites were coupled with *Blastocystis* carriage in the Thai, Turkish and South Korean sample sets, respectively. Of these, the majority were positively associated with the presence of *Blastocystis* in the gut. Between the countries, a total of 30 compounds were common to two or more, while dimethylamine and glycerol were elevated in all *B+* groups across the three countries. Of the identified metabolites that were increased, a number of them are usually obtained through diet and absorbed via the intestine [45,46]. Thus, the observed increase in the concentration of some of these compounds might in fact reflect inefficient absorption in the gut. *Blastocystis* has been previously hypothesised to increase intestinal permutability and inflammation [47], which in turn might impact intestinal function and the absorption of metabolites [48]. Nonetheless, it should be noted that there was an overall lack of metabolites typically associated with malabsorption including bile acids, amino acids and sphingolipids, as well as a significant decrease in short-chain fatty acids (SCFAs) [49,50,51]. Central among SCFAs is butyric acid, the preferential metabolic substrate of intestinal cells in the eubiotic gut [52,53]. In general, low amounts of SCFAs in the stool suggest their binding by gut receptors and use by enterocytes indicating a state of eubiosis [54]. Furthermore, other dysbiosis-associated compounds, such as inflammation-related metabolites were absent or decreased in *B+* individuals [55].

An inherent issue with faecal metabolomics is the non-homogeneity of faecal samples, unlike other bodily fluids, such as blood and cerebrospinal fluid. The faecal metabolome is influenced by numerous factors including diet and demographics [56]. In support of this, the PLS-DA plots cluster Thailand and South Korea together with Turkey displaying more dissimilarity in the variance (Supplementary Figure S3e). This possibly reflects the dietary similarities between the Asian countries compared to the more Mediterranean-style diet typically found in Turkey. Even with such disparity in samples, there was still a distinction between the samples from *B+* and *B−* individuals indicating a consistent pattern associated with *Blastocystis* presence. Within the groups, the tighter clustering of the *B+* samples indicates their metabolic homogeneity, while the same degree of clustering was not observed in *B−* samples, as they were much more heterogeneous. In this study, subtype data was not included, yet the lack of distinct groupings within the positive samples may indicate that the different subtypes are not having a profound impact on the metabolome of positive carriers. 

**Figure 4:**
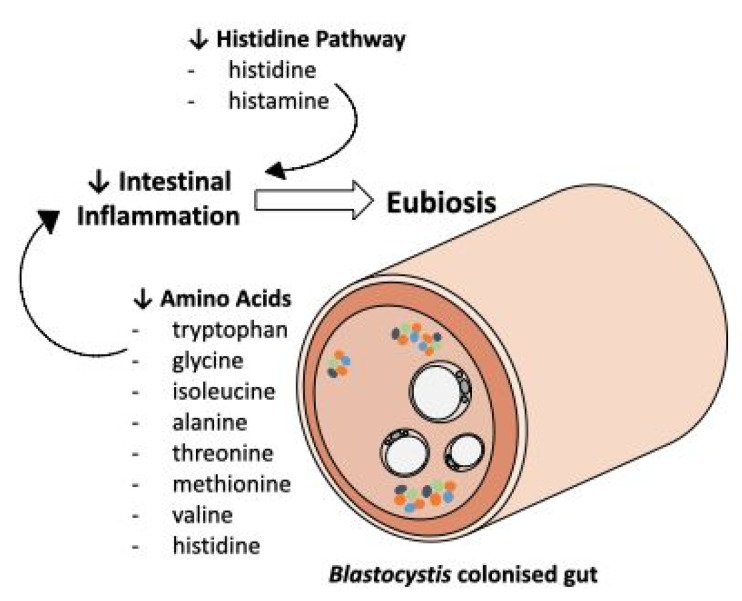
An overview of some of the commonly identified metabolites and pathways identified in this study investigating the associations between *Blastocystis* and the metabolome of humans. A decrease in a number of amino acids, in addition to a decrease in histidine pathway intermediates, may indicate that the presence of *Blastocystis* is a biomarker for a healthy gut.

To further identify prominent metabolites between *B+* and *B−,* variable importance in projection (VIP) was used, which correspond to the majority of significant metabolites identified by univariate analysis methods (Figure 2). Comparison of *B+* vs. *B−* VIP results across the entire dataset and between the *B+* and *B−* between each country, identified 20 common metabolites. Of these, eight were amino acids: Alanine, Glycine, Histidine, Isoleucine, Methionine, Threonine, Tryptophan and Valine, all of which are consistently decreased in the *B+* groups irrespective of country. This differential abundance of amino acids has also been observed in in-vivo studies using mice infected with the epicellular gut parasite *Cryptosporidium* [37,42]. Differences in amino acid concentrations were also found in a study conducted on *Cryptosporidium* in humans, but this investigation reported the converse, with a higher abundance of amino acids in infected individuals, which was likely due to malabsorption in the intestine [41]. The decrease of amino acids in *B+* individuals could indicate a protective/anti-inflammatory role of *Blastocystis* in the gut, as an increase of these metabolites is considered a biomarker for inflammation (Figure 4) [51,57].

Of the aforementioned amino acids, histidine, the precursor of histamine, is of special interest. Both histidine and histamine were significantly decreased across the *B+* samples. In the gut, histamine release is influenced by numerous factors including the host, diet and the overall microbiota composition. Its increase in the gut has also been noted in IBS and IBD sufferers [55,58,59]. The observed decrease in *B+* samples may link *Blastocystis* to anti-inflammatory responses in the gut. The mechanism for this is unknown, but perhaps the presence of *Blastocystis* is associated with bacterial communities that possess the ability to degrade histamine. Alternatively, *Blastocystis* colonisation might be associated with a reduction in bacterial communities that produce histamine. Nonetheless, these results demonstrate an apparent negative relationship between increased levels of histamine in the gut and the presence of *Blastocystis*.

It is worth noting that this is a pilot study and as such there are certain limitations. Samples were obtained from three different countries and despite following the same extraction protocol, consistency is not guaranteed. Moreover, while the Thai and Turkish samples were obtained and extracted a couple of hours after collection (stored at 4 °C), South Korean samples were frozen at −20 °C for a short amount of time before extraction, potentially affecting the metabolites detected. Finally, groups from different countries ranged from asymptomatic to those with GI symptoms, however, this was considered and analyses removing particular groups were carried out to account for their effect. Due to the nature and objectives of this study, we are not able to speculate or draw conclusions regarding the roles of *Blastocystis* in the host-microbiome-immunomodulation axis. In vitro studies on *Blastocystis* subtype 7 have demonstrated the release of proteases with antibody-degrading activity [20,60,61,62], which could affect the presence and concentration of amino acids in the gut. Considering all of the above, the current work provides a baseline of metabolites present in *B+* human hosts and will be essential in future studies investigating the microbial composition and metabolic profiling at the level of subtype and beyond.

## 4. Materials and Methods

### 4.1. Ethical Approval

The ethics committee of Mae Fah Luang University (Chiang Rai, Thailand) approved the collection of human samples from Thailand used in this study (human license approval number REH60103). Ethical rules were in accordance to the Declaration of Helsinki. Data were strictly anonymised and assigned codes.

The IRB corresponding to the collection of samples from South Korea was CNUHH-2020-045.

The Gazi University Ethic Commission (Ankara, Turkey) approved the collection of human samples from Turkey for this study (code; 2017-248). 

### 4.2. Sample Collection

Faecal samples were collected from a total of 149 individuals from three countries. Of these, 40 were from South Korea, 43 from Thailand and 71 were from Turkey (Supplementary Table S1). Details of the collections were as follows.

South Korea: the samples used herein are part of an already published study [63]. All samples were collected from hospitals. Twenty samples were collected from diarrhetic individuals, all of whom were negative for *Blastocystis* and 20 were from non-diarrhetic volunteers positive for *Blastocystis*.

Thailand: samples were collected from individuals living in Chiang Rai Province. Volunteers did not suffer from gastrointestinal diseases and displayed no gastrointestinal-related symptoms at the time of collection. 

Turkey: all 71 samples were obtained from diarrhetic volunteers who underwent checkups in the hospital.

### 4.3. Blastocystis Detection

South Korea: Samples were surveyed using molecular detection as described previously [63].

Thailand: samples were surveyed using qPCR to amplify a fragment of the SSUrRNA gene according to previously published protocols [64]. The primers used for the reactions were: forward BL18SPPF1; 5′-AGTAGTCATACGCTCGTCTCAAA-3′) and reverse (BL18SR2PP; 5′-TCTTCGTTACCCGTTACTGC-3′.

Turkey: samples were surveyed using qPCR to amplify a fragment of the SSUrRNA gene according to previously published protocols [65].

Collected samples were stored in sterile collection tubes at 4 °C until metabolite extraction (no longer than a couple of hours) in the case of Thailand and Turkey. Samples were frozen at −20 °C in the case of South Korea.

### 4.4. Sample Preparation

Metabolites were extracted after stool collection in the case of Thailand and Turkey, and from frozen samples in the case of South Korea. The extraction protocol was as follows: 200 mg of dried faecal sample was added to 6 mL of 75% analytical grade ethanol at 80 °C (Fisher) and 200 mg of 2 mm diameter glass beads. The sample was then agitated via vortexing for 30 s. The mixture was incubated at 80 °C for 3 min and agitated further until the sample was completely homogenised. The homogenised sample was aliquoted into three 2 mL sterile tubes (Eppendorf) and centrifuged at 16,000× *g* for 10 min at room temperature. The supernatant was transferred to new sterile 2 mL tubes. The samples were dried overnight in a rotorvac at 40 °C. The dried sample was suspended in 330 μL double distilled water and vortexed at maximum speed for 30 s until the desiccate was fully dissolved before being centrifuged at 2500× *g* for 10 min at room temperature. The supernatant of each sample was combined into a sterile 1.5 mL microcentrifuge tube (Eppendorf) and stored at −80 °C until analysis.

650 μL of sample for analysis was mixed with 35 μL of a premixed stock of deuterium oxide (D_2_O) (CortecNet) and 10 mM Sodium trimethylsilylpropanesulfonate (DSS-d6) (Sigma) to a final concentration of 0.5 mM. The pH of each sample was recorded (LAQUA twin pH meter, Qiagen) before being added to a 535-PP7 NMR tubes (Wilmad).

### 4.5. NMR Spectroscopy

Samples were analysed using one-dimensional (1D) ^1^H NMR spectroscopy on a 600 MHz AVANCE III spectrometer equipped with a QCI-P cryoprobe (Bruker) at 298 K with a transmitter frequency of 600.05 MHz locked to D_2_O (5% *v*/*v*). Tuning and shimming were carried out automatically for each sample as was the 90° pulse calibration. The receiver gain was limited to a maximum value of 128. Data used for metabolite concentration analysis were drawn from presat-NOESY (noesygppr1d) spectra with a mix time (T_mix_) of 100 ms using industry standard parameters compatible parameters compatible with the Chenomx metabolite library (CHENOMX 9.0 https://www.chenomx.com; accessed on: 1 June 2019). The presat-NOESY was measured using 512 scans and 8 dummy scans with a spectral width of 15.98 ppm (9590.75 Hz), giving an acquisition time of 1.71 s, a relaxation delay of 3 was used and the data size was 32,768 points giving a total recycle time of 4.7 s. A second excitation sculpting experiment was carried out for quality control with 256 scans and 8 dummy scans; the spectral width was 15.98 ppm (9590.79 Hz), acquisition time 1.7 s and relaxation delay 3 s; with a data size of 32,768 data points. For all experiments, the water resonance was optimised (automatically) for maximum suppression (o1p was ~4.699 ppm), the field strength of the presaturation was 140.25 Hz (standard bruker parameters) and presaturation was applied both during the D1 delay and the mixing T_mix_. To avoid systematic errors samples were prepared and measured in random order.

### 4.6. Data Processing and Statistical Analysis

#### 4.6.1. Analysis of Spectra

The resulting spectra were phased, manually baseline corrected and line-broadened with a 1 Hz exponential window function in TOPSPIN 4.0.9 (Bruker). Processed spectra were imported into Chenomx 8.4, where the Chenomx Processor was used to perform standard profiling and calibration. The region between 4.56 ppm to 4.97 ppm was deleted to eliminate the H2O resonance peak. pH adjustments were made based on the recorded pH of the samples. In Chenomx peak assignment, identification and quantification was achieved using the Chenomx profiler tool, spectra were automatically fitted to the 338 reference compounds in the Chenomx library, each spectrum was manually checked and adjusted to account for the non-homeostatic nature (varying pH and metal ion content) of faecal samples before the resulting compound concentrations were exported. Good agreement between individual simulated metabolite spectra from the Chenomx library and the measured data were achieved, with attention to complete fits across the peaks from a metabolite to the total spectra envelope being required for positive identification. Sample concentrations were measured relative to the internal DSS-d6 standard.

Exported compound concentrations were input into the MetaboAnalyst 5.0 pipeline as one-factor, unpaired data [66] (https://www.metaboanalyst.ca; accessed on: 15 March 2021) for statistical analysis and visual plot production.

#### 4.6.2. Grouping and Statistical Analysis

Data was grouped in nine different ways considering the presence of *Blastocystis*, country of origin and whether volunteers were diarrhetic. The human faecal metabolite profiles were analysed based on all groupings with a special focus on *Blastocystis* positive (*B+*) vs. negative (control group) (*B−*) per country. The results for each country were analysed independently from each other to reduce variability as well as compiled together to investigate associations of *Blastocystis* colonisation across the sample sets.

The statistical analysis was performed using MetaboAnalyst 5.0. Data filtering by the mean of intensity values was implemented to remove non-informative results, including compound values, which were close to the baseline. Sample normalization was applied to remove undesired systematic biases to retain biologically relevant differences in the data. Normalization was applied using the DSS chemical shift indicator to account for any differences in reference concentrations between samples, and when combined analysis of different countries was carried out (reference DSS concentrations were 0.5 mM or 1 mM depending on sample cohort). Data scaling was carried out by the auto-scaling method to help balance signal intensity variances, which originate from differences in the average abundance of metabolites [67]. Before statistical analysis, the overall data quality of metabolite concentration values was checked for any visually obvious outliers.

Univariate analysis of metabolite profiles obtained from seven of the nine groups (Table 1, excluding country and country and *Blastocystis* status groupings) was used to determine which metabolites were different between the two groups based on the fol*D*−change (FC) values that exceeded the threshold of significance (set at 2) and Wilcoxon rank-sum tests (*p* < 0.05). Data distribution was checked using the Shapiro–Wilk test for normality. Analysis was carried out on false discovery rate (FDR) adjusted *p*-values (based on Benjamini–Hochberg procedure) for multiple comparisons approach with a *p*-value of < 0.05.

Multivariate analysis was carried out on all nine groups to investigate patterns between *Blastocystis* carriage and resulting metabolite profiles in comparison to uninfected controls. This was implemented using unsupervised Principal Component Analysis (PCA). Supervised analysis based on Partial Least-Squares Discriminant Analysis (PLS-DA) was also applied to the dataset in order to investigate the prediction of differences in the metabolite profiles in the various groups. PLS-DA analysis was cross-validated using the leave-one-out cross-validation (LOOCV) method to address the issue of possible overfitting.

Pathway enrichment analysis was carried out on identified metabolites to determine if differences exist between metabolic pathways in *Blastocystis* positive and negative individuals. This was implemented by assessing normalised metabolite concentrations via MetPA’s (Metabolomics Pathway Analysis) pathway topology analysis [68,69] available on the MetaboAnalyst 5.0 pipeline.

## 5. Conclusions

This is the first study of its kind focusing on *Blastocystis* and the gut metabolome. The overall reduction of inflammation-related metabolites suggests an anti-inflammatory role of *Blastocystis* in the gut (Figure 4). Despite this, we cannot exclude the inflammatory roles of specific subtypes. Further in vitro and in vivo investigations on profiling the overall microbiome in association with the metabolome would greatly contribute towards elucidating the interactions of *Blastocystis* in the gut.

## Figures and Tables

**Table 1:** Stating the different variables, their notations and sample number included in this study. Symptomatic South Korean samples were often excluded due to other diagnosed pathologies. Hence the exclusion of these samples in some analyses.

Variable	Notation	No. Groups	No. Samples
Thailand	*B*+/*B*−	2	44
Turkey	*B*+/*B*−	2	69
South Korea	*B*+/*B*−	2	38
Positive vs. Negative	*B*+/*B*−	2	151
Asymptomatic *Blastocystis* positive vs. Symptomatic, *Blastocystis* negative	*D*−*B*+/*D*+*B*−	2	98
Asymptomatic, *Blastocystis* positive vs. Asymptomatic, *Blastocystis* negative	*D*−*B*+/*D*−*B*−	2	62
*Blastocystis* positive vs. *Blastocystis* negative (excluding *Blastocystis* negative from Korea)	*B*+/*B*−	2	131
Country and *Blastocystis* status	Country *B*+/*B*−	6	151
Country	Turkey/Thailand/S. Korea	3	151

## Data Availability

The data presented in this study are available in article.

## Supplementary Materials

The following are available online at https://www.mdpi.com/article/10.3390/metabo11120883/s1. Table S1: Showing samples used in this study including the country or origin, *Blastocystis* carriage status and the type of stool sample collected which relates to symptomology, Table S2: Significant metabolites identified across the three country cohorts. Those in green represent metabolites that were significantly increased in B+ samples as identified by univariate analysis. Those in blue were significantly decreased in the B+ samples when compared against B−. 5/30 metabolites had different expression results between at least two of the cohorts, Table S3: Listing all 98 significant metabolites identified from the B+ vs. B− sample cohort that were above the log2 fold change threshold. Univariate analysis was carried out with non-parametric tests on FDR-adjusted values (p-adj 0.05), Table S4: Listing all 166 significant metabolites identified from the D−B+ vs. D+B− sample cohort that were above the log2 fold change threshold. Univariate analysis was carried out with non-parametric tests on FDR-adjusted values (p-adj 0.05), Table S5: Listing all six significant metabolites identified from the D−B+ vs. D−B− sample cohort that were above the log2 fold change threshold. Univariate analysis was carried out with non-parametric tests on FDR-adjusted values (p-adj 0.05), Table S6: Listing all 51 significant metabolites identified from the B+(D+ and D−) excluding Korea D+ sample cohort that were above the log2 fold change threshold. Univariate analysis was carried out with non-parametric tests on FDR-adjusted values (p-adj 0.05), Table S7: Details of pathway analysis for the Thai cohort, only values with p-adjust <0.05 are included. The ‘Total’ column equates to the total number of compounds in the given pathway and the ‘Hits’ are compounds from this dataset that match to a given pathway. The p-adjust values are adjusted Raw *p*-values following the Holm-Bonferroni method. FDR is false discovery rate adjusted *p*-value and the Impact is the impact value was calculated from pathway topology analysis, Table S8: Details of pathway analysis for the Turkey cohort, only values with p-adjust <0.05 are included. The ‘Total’ column equates to the total number of compounds in the given pathway and the ‘Hits’ are compounds from this dataset that match to a given pathway. The p-adjust values are adjusted Raw *p*-values following the Holm-Bonferroni method. FDR is false discovery rate adjusted *p*-value and the Impact is the impact value was calculated from pathway topology analysis, Table S9: Details of pathway analysis for the South Korean cohort, only values with p-adjust <0.05 are included. The ‘Total’ column equates to the total number of compounds in the given pathway and the ‘Hits’ are compounds from this dataset that match to a given pathway. The p-adjust values are adjusted Raw *p*-values following the Holm-Bonferroni method. FDR is false discovery rate adjusted *p*-value and the Impact is the impact value was calculated from pathway topology analysis, Table S10: Details of pathway analysis for the B+ vs. B− samples, only values with p-adjust <0.05 are included. The ‘Total’ column equates to the total number of compounds in the given pathway and the ‘Hits’ are compounds from this dataset that match to a given pathway. The p-adjust values are adjusted Raw *p*-values following the Holm-Bonferroni method. FDR is false discovery rate adjusted *p*-value and the Impact is the impact value was calculated from pathway topology analysis, Figure S1: Example 1D 1H NMR spectra from each region in B− and B+ individuals. (a) Thailand B− (b) Thailand B+ (c) South Korea B− (d) South Korea B+ (e) Turkey B− (f) Turkey B+, Figure S2: Multivariate analysis showing Unsupervised principal component analysis (PCA) of the normalized metabolite concentrations (a) Thailand, (b) Turkey, (c) South Korea, (d) B+ vs. B−, (e) D−B+ vs. D+B−, (f) D−B+ vs. D−B−, (g) B+ vs. B− (Korea B− excluded), (h) Country & *Blastocystis*, (i) Country, Figure S3: Multivariate analysis showing Partial Least Squares Discriminant Analysis (PLS-DA) (a) D−B+ vs. D+B−, (b) D−B+ vs. D−B−, (c) B+ vs. B− (Korea B− excluded), (d) Country & *Blastocystis*, (e) Country, Figure S4: VIP score plot summarizing the 30 most important metabolites determined by the PLS-DA plot. The x-axis indicates the VIP score corresponding to significant metabolites on the y-axis. The coloured boxes on the right represent the relative concentration of the metabolite. (a) D−B+ vs. D+B−, (b) D−B+ vs. D−B−, (c) B+ vs. B− (Korea B− excluded), (d) Country & *Blastocystis*, (e) Country.
