# Supplementary material for: Metabolic Fluctuations in the Human Stool Obtained from Blastocystis Carriers and Non-Carriers
Source: Metabolites. 2021 Dec 17;11(12):883. doi: 10.3390/metabo11120883 (PMC8708241; doi:10.3390/metabo11120883)
Supplement: Supplementary file 1 [file metabolites-11-00883-s001.zip › Supplementary Figures.pdf]

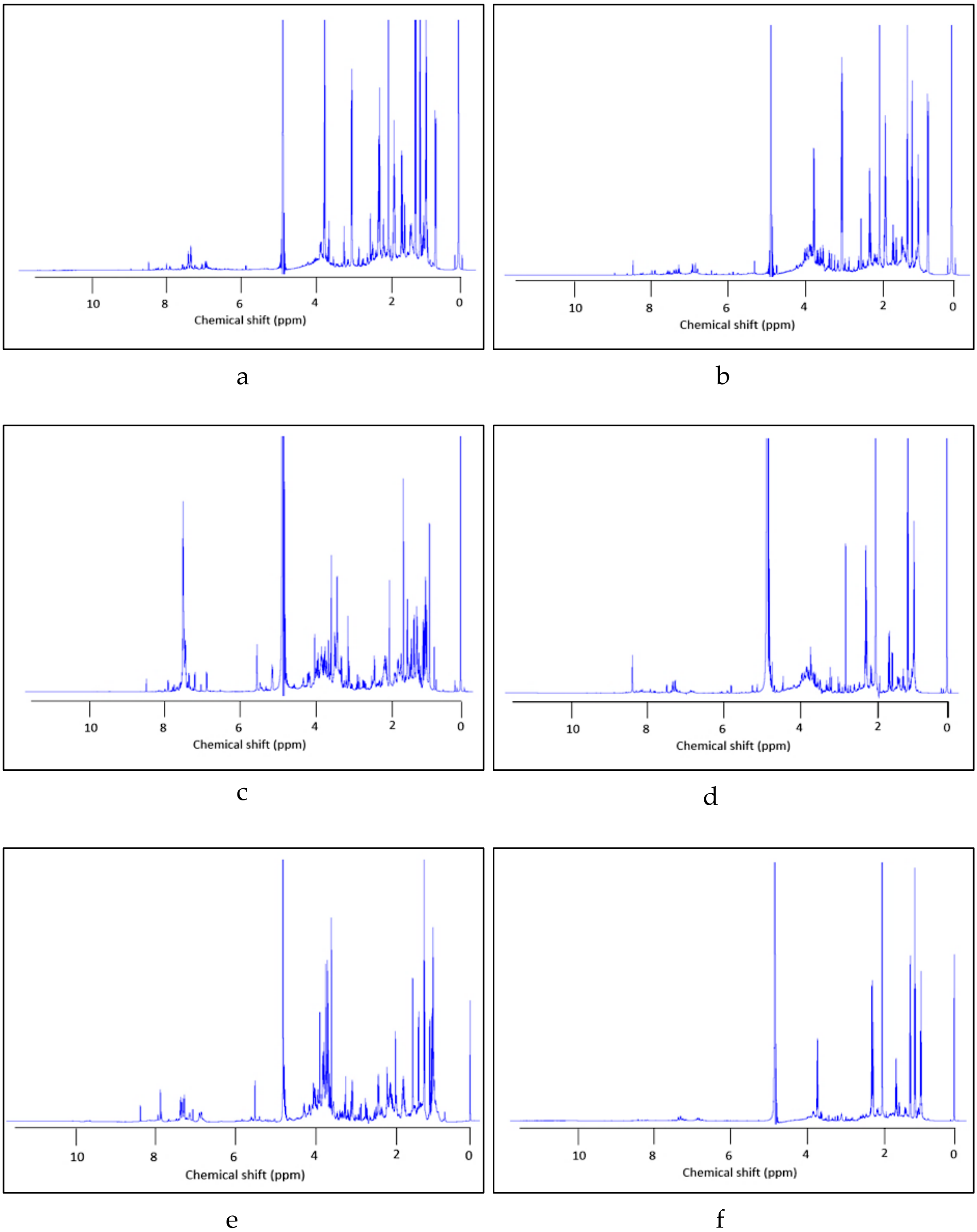

Figure S1. Example 1D 1H NMR spectra from each region in B- and B+ individuals. *a*) Thailand *B-* *b*) Thailand *B+* *c*) South Korea *B-* *d*) South Korea *B+* *e*) Turkey *B-* *f*) Turkey *B+*

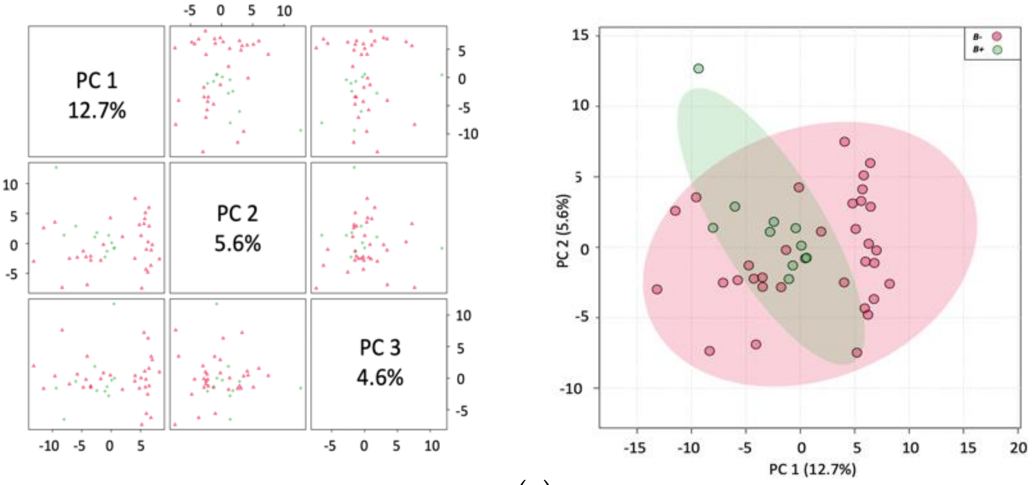
PC 1
12.7%
PC 2
5.6%
PC 3
4.6%
B-
B+
PC 2 (5.6%)
PC 1 (12.7%)

(a)

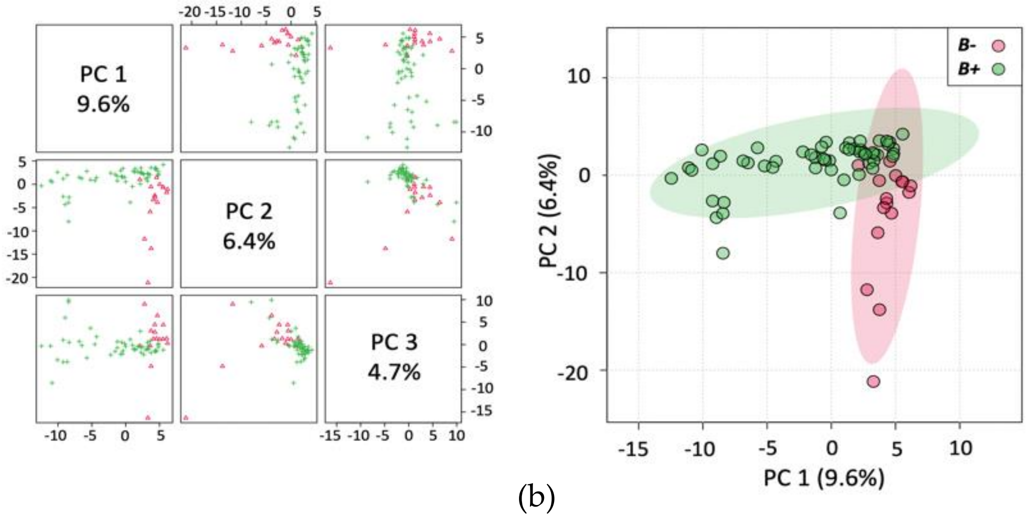
PC 1
9.6%
PC 2
6.4%
PC 3
4.7%
B-
B+
PC 2 (6.4%)
PC 1 (9.6%)

(b)

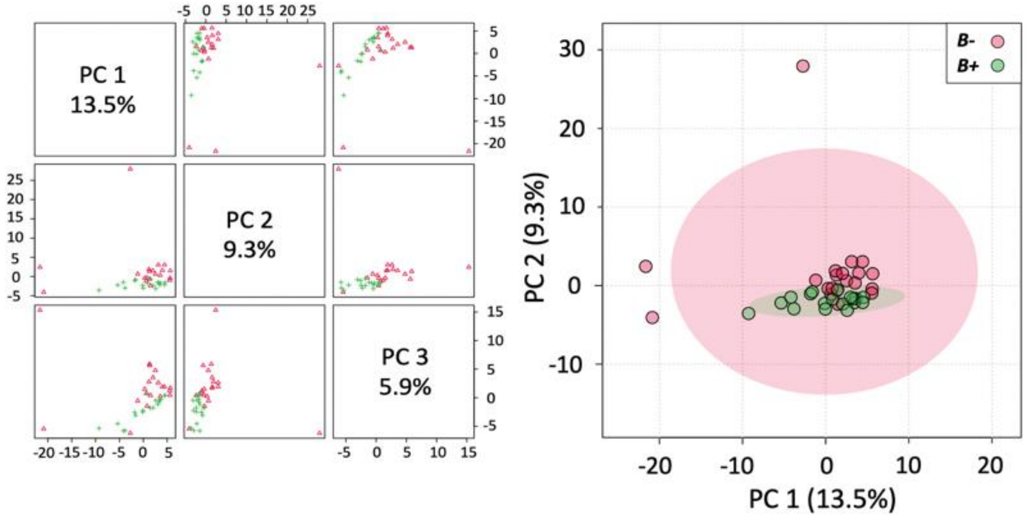
PC 1
13.5%
PC 2
9.3%
PC 3
5.9%
B-
B+
PC 2 (9.3%)
PC 1 (13.5%)

(c)

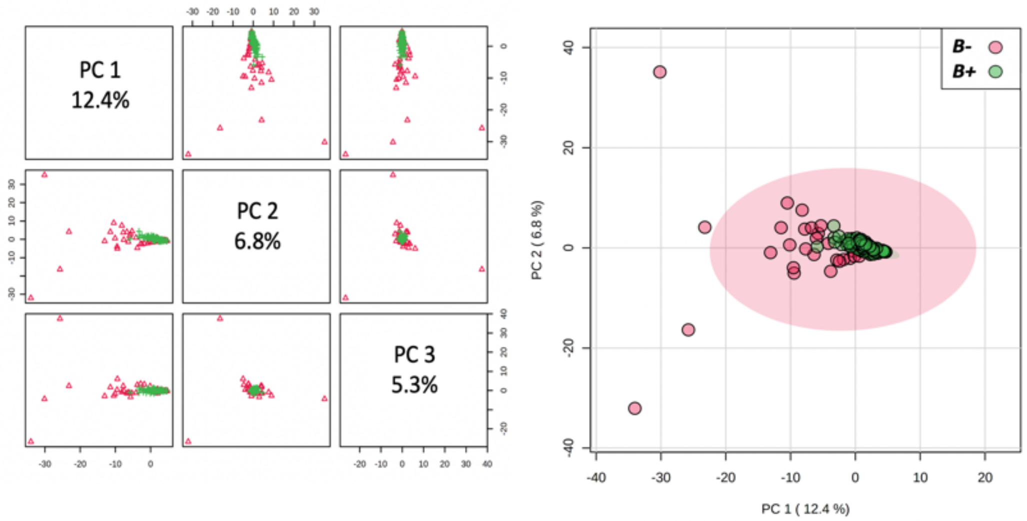
PC 1
12.4%
PC 2
6.8%
PC 3
5.3%
B-
B+
PC 2 (6.8 %)
PC 1 (12.4 %)

(d)

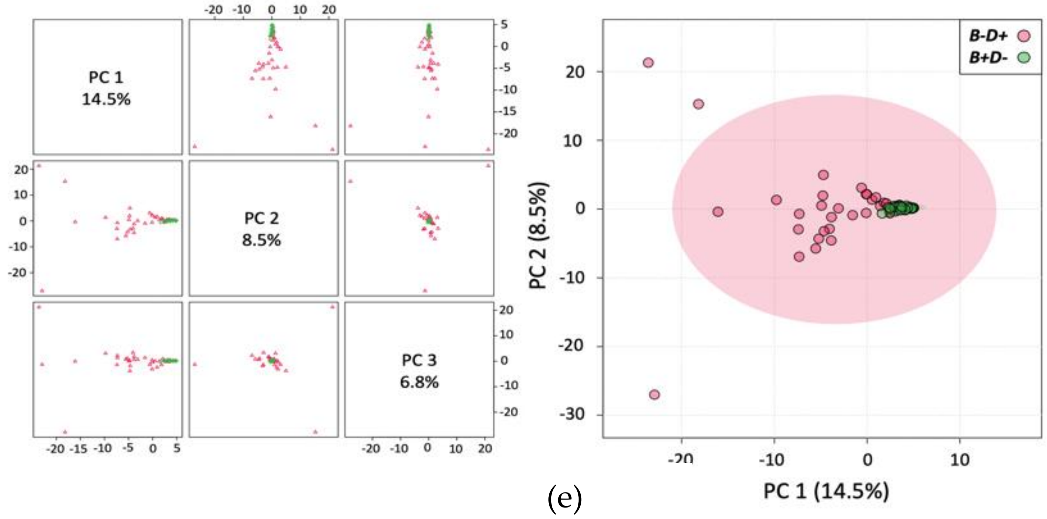
PC 1
14.5%
PC 2
8.5%
PC 3
6.8%
B-D+
B+D-
PC 2 (8.5%)
PC 1 (14.5%)

(e)

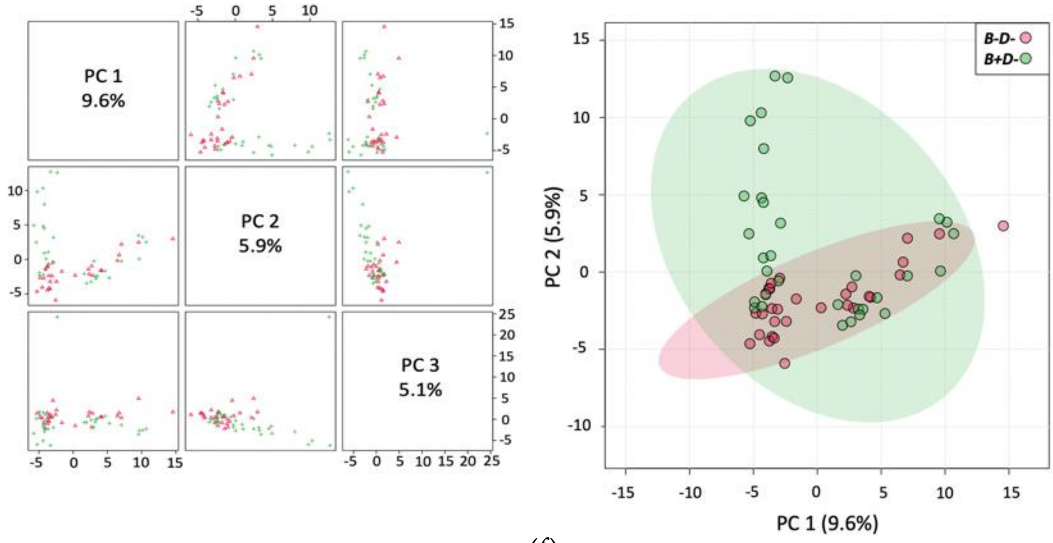
PC 1
9.6%
PC 2
5.9%
PC 3
5.1%
B-D-
B+D-
PC 2 (5.9%)
PC 1 (9.6%)

(f)

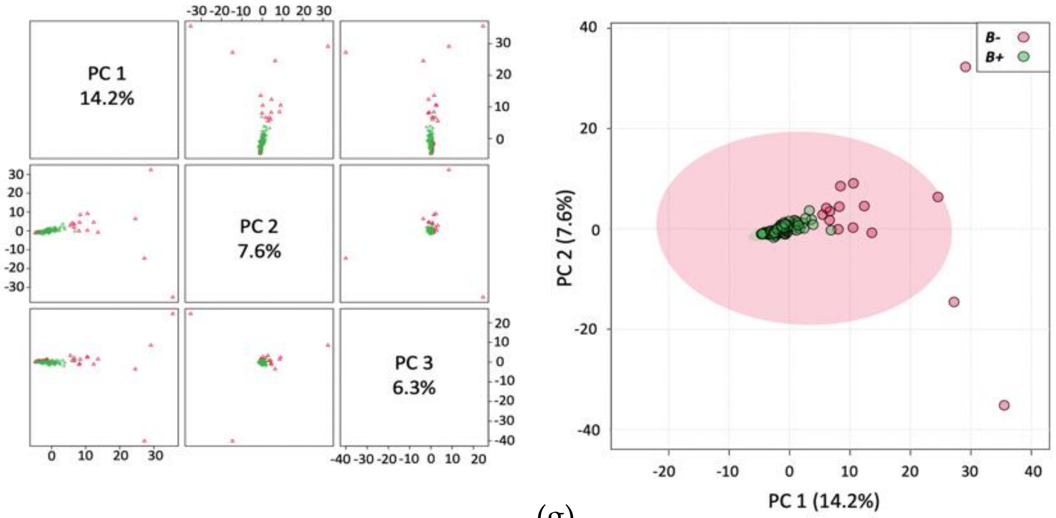

(g)

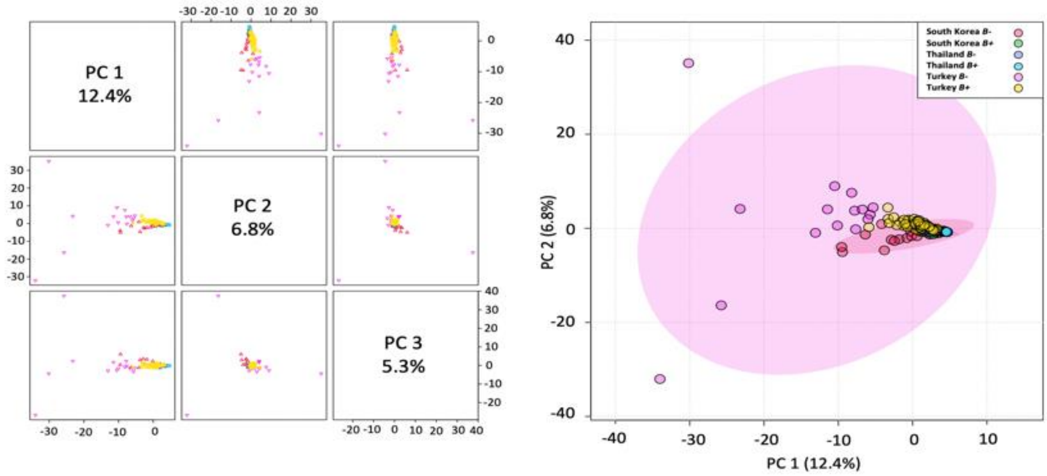

(h)

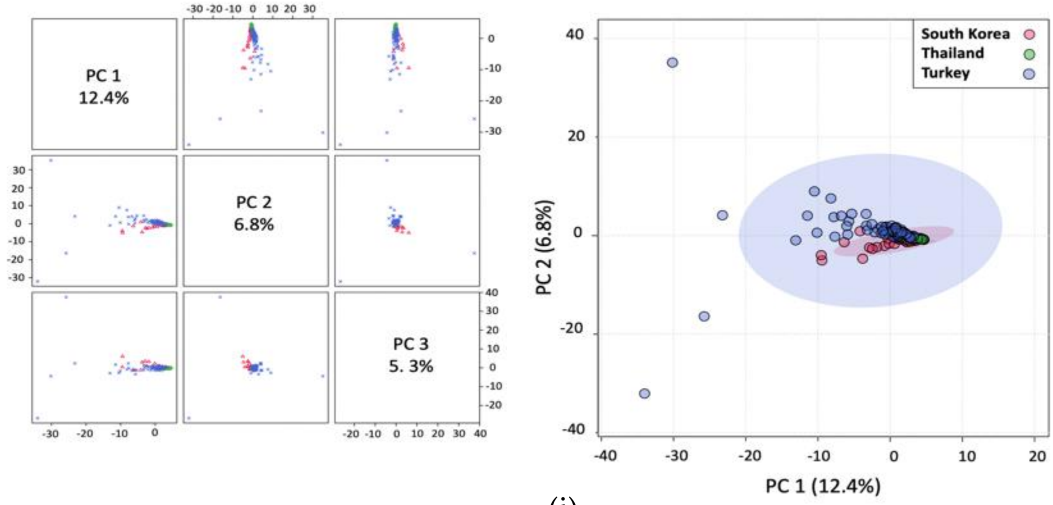

(i)

Figure S2. Multivariate analysis showing Unsupervised principal component analysis (PCA) of the normalized metabolite concentrations *a)* Thailand, *b)* Turkey, *c)*

South Korea, *d)* B+ vs B-, *e)* D-B+ vs D+B-, *f)* D-B+ vs D-B-, *g)* B+ vs B- (Korea B- excluded), *h)* Country & *Blastocystis*, *i)* Country.

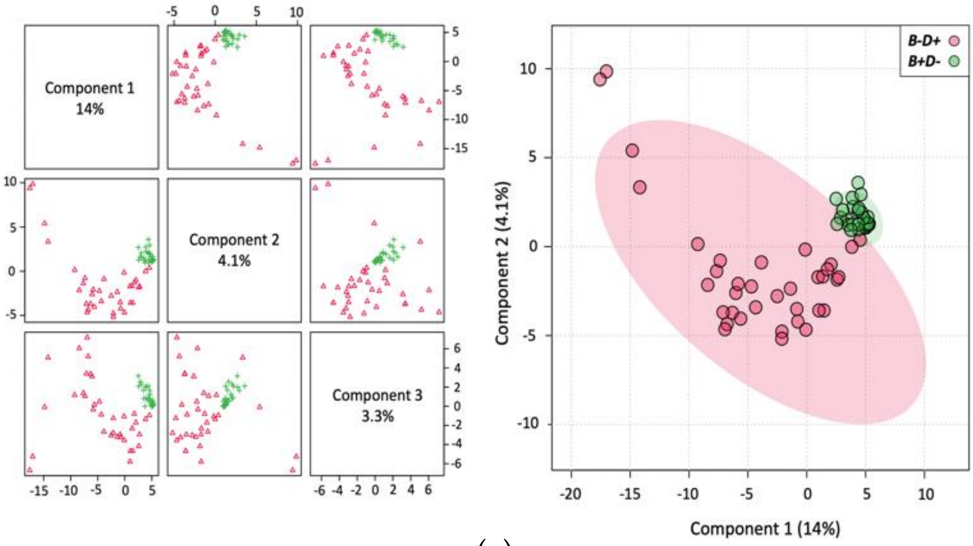
Component 1
14%
Component 2
4.1%
Component 3
3.3%
B-D+
B+D-
Component 2 (4.1%)
Component 1 (14%)

(a)

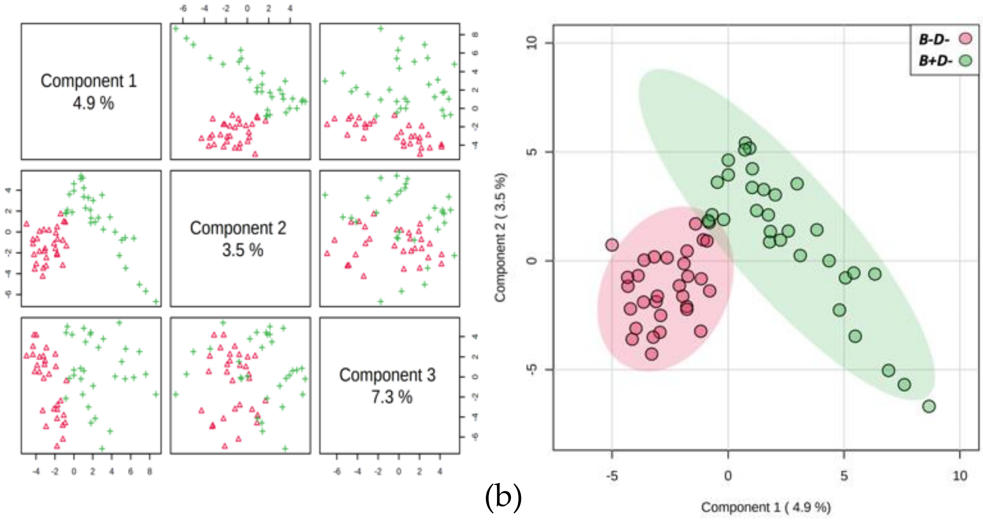
Component 1
4.9 %
Component 2
3.5 %
Component 3
7.3 %
B-D-
B+D-
Component 2 (3.5 %)
Component 1 (4.9 %)

(b)

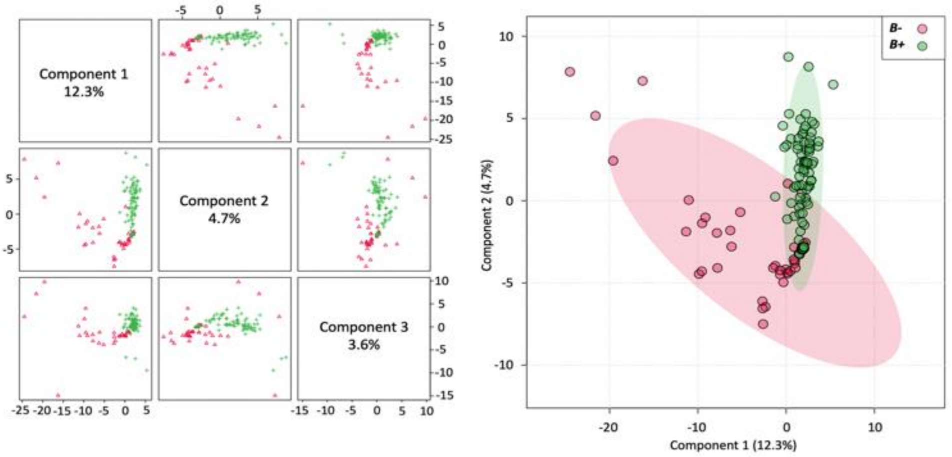
Component 1
12.3%
Component 2
4.7%
Component 3
3.6%
B-
B+
Component 2 (4.7%)
Component 1 (12.3%)

(c)

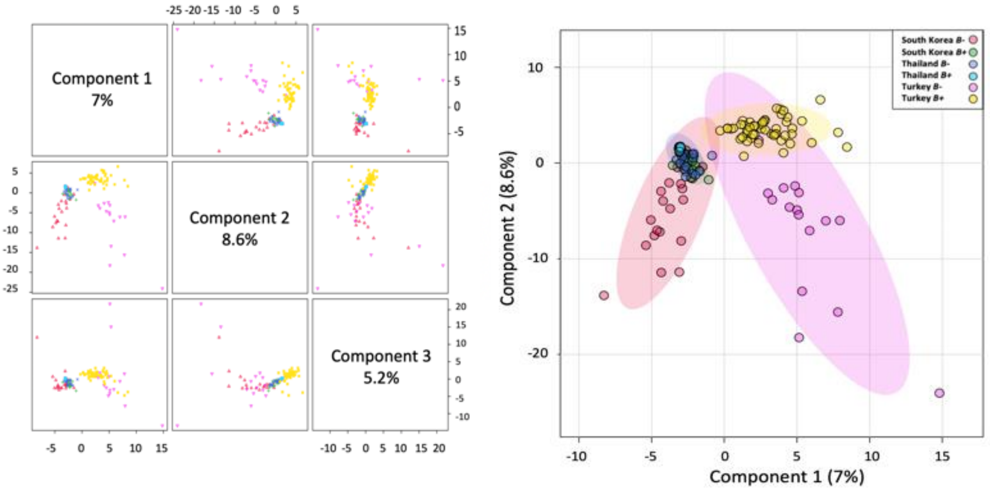

(d)

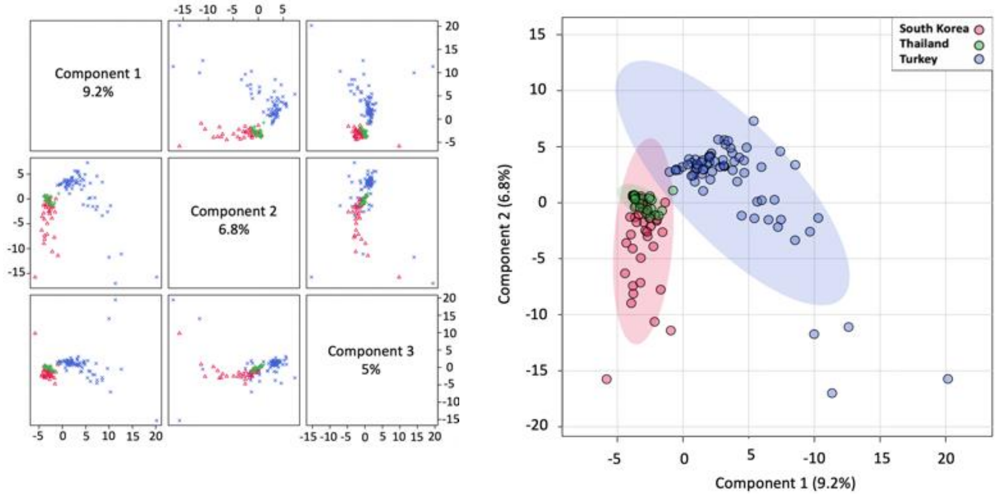

(e)

Figure S3. Multivariate analysis showing Partial Least Squares Discriminant Analysis (PLS-DA) *a)* D-B+ vs D+B-, *b)* D-B+ vs D-B-, *c)* B+ vs B- (Korea B- excluded), *d)* Country & *Blastocystis*, *e)* Country

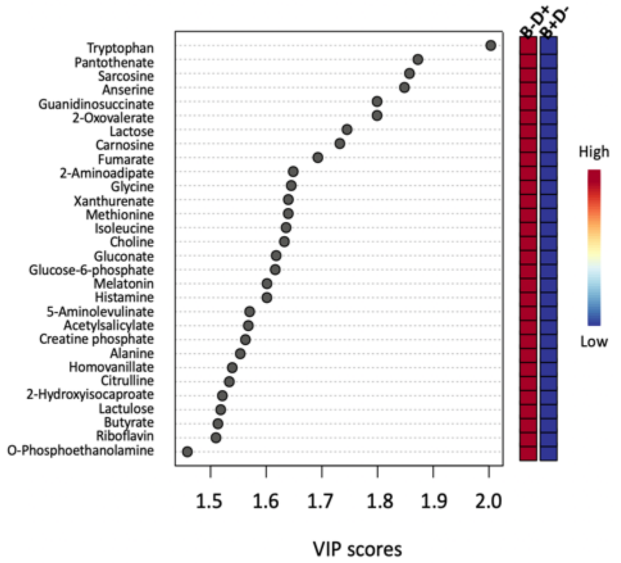

B- D+
B+ D-
Tryptophan
Pantothenate
Sarcosine
Anserine
Guanidinosuccinate
2-Oxovalerate
Lactose
Carnosine
Fumarate
2-Aminoadipate
Glycine
Xanthurenate
Methionine
Isoleucine
Choline
Gluconate
Glucose-6-phosphate
Melatonin
Histamine
5-Aminolevulinate
Acetylsalicylate
Creatine phosphate
Alanine
Homovanillate
Citrulline
2-Hydroxyisocaproate
Lactulose
Butyrate
Riboflavin
O-Phosphoethanolamine
High
Low
1.5 1.6 1.7 1.8 1.9 2.0
VIP scores

(a)

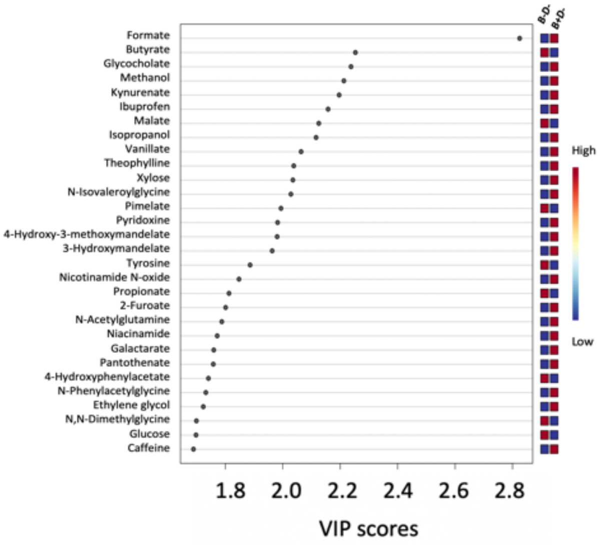

B-D-
B+D-
Formate
Butyrate
Glycocholate
Methanol
Kynurenate
Ibuprofen
Malate
Isopropanol
Vanillate
Theophylline
Xylose
N-Isovaleroylglycine
Pimelate
Pyridoxine
4-Hydroxy-3-methoxymandelate
3-Hydroxymandelate
Tyrosine
Nicotinamide N-oxide
Propionate
2-Furoate
N-Acetylglutamine
Niacinamide
Galactarate
Pantothenate
4-Hydroxyphenylacetate
N-Phenylacetylglycine
Ethylene glycol
N,N-Dimethylglycine
Glucose
Caffeine
High
Low
1.8 2.0 2.2 2.4 2.6 2.8
VIP scores

(b)

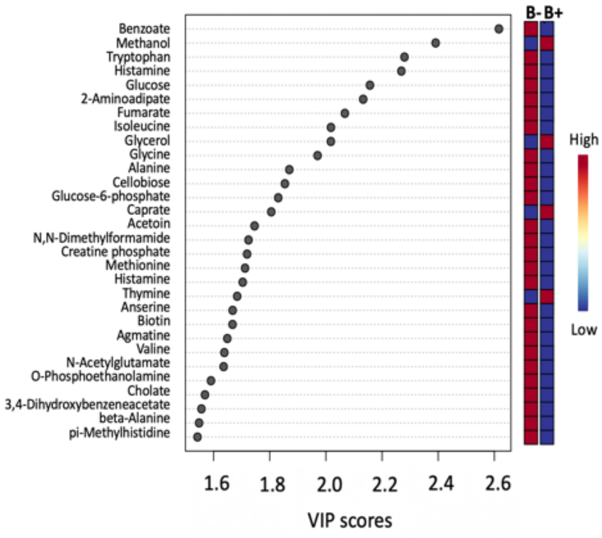

B-
B+
Benzoate
Methanol
Tryptophan
Histamine
Glucose
2-Aminoadipate
Fumarate
Isoleucine
Glycerol
Glycine
Alanine
Cellobiose
Glucose-6-phosphate
Caprate
Acetoin
N,N-Dimethylformamide
Creatine phosphate
Methionine
Histamine
Thymine
Anserine
Biotin
Agmatine
Valine
N-Acetylglutamate
O-Phosphoethanolamine
Cholate
3,4-Dihydroxybenzeneacetate
beta-Alanine
pi-Methylhistidine
High
Low
1.6 1.8 2.0 2.2 2.4 2.6
VIP scores

(c)

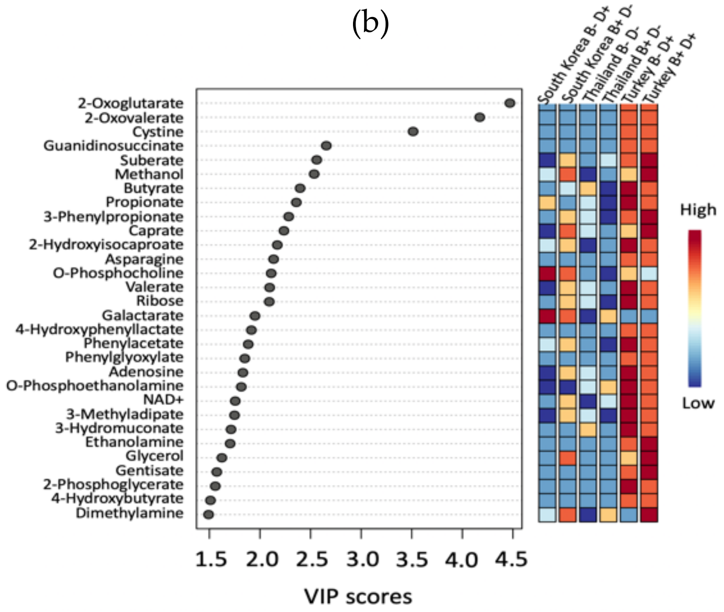

South Korea B- D+
South Korea B+ D-
Thailand B- D-
Thailand B+ D-
Turkey B- D+
Turkey B+ D+
2-Oxoglutarate
2-Oxovalerate
Cystine
Guanidinosuccinate
Suberate
Methanol
Butyrate
Propionate
3-Phenylpropionate
Caprate
2-Hydroxyisocaproate
Asparagine
O-Phosphocholine
Valerate
Ribose
Galactarate
4-Hydroxyphenyllactate
Phenylacetate
Phenylglyoxylate
Adenosine
O-Phosphoethanolamine
NAD+
3-Methyladipate
3-Hydromuconate
Ethanolamine
Glycerol
Gentisate
2-Phosphoglycerate
4-Hydroxybutyrate
Dimethylamine
High
Low
1.5 2.0 2.5 3.0 3.5 4.0 4.5
VIP scores

(d)

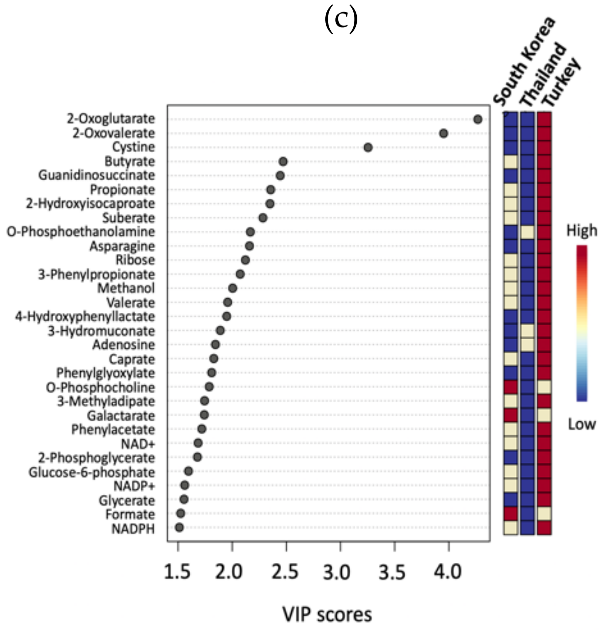

South Korea
Thailand
Turkey
2-Oxoglutarate
2-Oxovalerate
Cystine
Butyrate
Guanidinosuccinate
Propionate
2-Hydroxyisocaproate
Suberate
O-Phosphoethanolamine
Asparagine
Ribose
3-Phenylpropionate
Methanol
Valerate
4-Hydroxyphenyllactate
3-Hydromuconate
Adenosine
Caprate
Phenylglyoxylate
O-Phosphocholine
3-Methyladipate
Galactarate
Phenylacetate
NAD+
2-Phosphoglycerate
Glucose-6-phosphate
NADP+
Glycerate
Formate
NADPH
High
Low
1.5 2.0 2.5 3.0 3.5 4.0
VIP scores

(e)

Figure S4. VIP score plot summarizing the 30 most important metabolites determined by the PLS-DA plot. The x-axis indicates the VIP score corresponding to significant metabolites on the y-axis. The coloured boxes on the right represent the relative concentration of the metabolite. *a)* D-B+ vs D+B-, *b)* D-B+ vs D-B-, *c)* B+ vs B- (Korea B- excluded), *d)* Country & *Blastocystis*, *e)* Country
