# Supplementary material for: Metabolic Fluctuations in the Human Stool Obtained from Blastocystis Carriers and Non-Carriers
Source: Metabolites. 2021 Dec 17;11(12):883. doi: 10.3390/metabo11120883 (PMC8708241; doi:10.3390/metabo11120883)
Supplement: Supplementary file 1 [file metabolites-11-00883-s001.zip › Supplementary Tables.pdf]

Table S1. Showing samples used in this study including the country or origin, *Blastocystis* carriage status and the type of stool sample collected which relates to symptomology

Sample ID	Country of origin	*Blastocystis* infection status	Stool type
**MB/BF 1**	Turkey	Positive	Diarrheal
**MB/BF 2**	Turkey	Positive	Diarrheal
**MB/BF 3**	Turkey	Positive	Diarrheal
**MB/BF 4**	Turkey	Positive	Diarrheal
**MB/BF 5**	Turkey	Positive	Diarrheal
**MB/BF 6**	Turkey	Positive	Diarrheal
**MB/BF 7**	Turkey	Positive	Diarrheal
**MB/BF 8**	Turkey	Positive	Diarrheal
**MB/BF 9**	Turkey	Positive	Diarrheal
**MB/BF 10**	Turkey	Positive	Diarrheal
**MB/BF 11**	Turkey	Positive	Diarrheal
**MB/BF 12**	Turkey	Positive	Diarrheal
**MB/BF 13**	Turkey	Positive	Diarrheal
**MB/BF 14**	Turkey	Positive	Diarrheal
**MB/BF 17**	Turkey	Positive	Diarrheal
**MB/BF 18**	Turkey	Positive	Diarrheal
**MB/BF 19**	Turkey	Positive	Diarrheal
**MB/BF 20**	Turkey	Positive	Diarrheal
**MB/BF 21**	Turkey	Positive	Diarrheal
**MB/BF 22**	Turkey	Positive	Diarrheal
**MB/BF 23**	Turkey	Positive	Diarrheal
**MB/BF 24**	Turkey	Positive	Diarrheal
**MB/BF 25**	Turkey	Positive	Diarrheal

**MB/BF 26**	Turkey	Positive	Diarrheal
**MB/BF 27**	Turkey	Positive	Diarrheal
**MB/BF 28**	Turkey	Positive	Diarrheal
**MB/BF 29**	Turkey	Positive	Diarrheal
**MB/BF 30**	Turkey	Positive	Diarrheal
**MB/BF 31**	Turkey	Positive	Diarrheal
**MB/BF 32**	Turkey	Positive	Diarrheal
**MB/BF 33**	Turkey	Positive	Diarrheal
**MB/BF 34**	Turkey	Positive	Diarrheal
**MB/BF 35**	Turkey	Positive	Diarrheal
**MB/BF 36**	Turkey	Positive	Diarrheal
**MB/BF 37**	Turkey	Positive	Diarrheal
**MB/BF 38**	Turkey	Positive	Diarrheal
**MB/BF 39**	Turkey	Positive	Diarrheal
**MB/BF 40**	Turkey	Positive	Diarrheal
**MB/BF 41**	Turkey	Positive	Diarrheal
**MB/BF 42**	Turkey	Positive	Diarrheal
**MB/BF 43**	Turkey	Positive	Diarrheal
**MB/BF 44**	Turkey	Positive	Diarrheal
**MB/BF 45**	Turkey	Positive	Diarrheal
**MB/BF 46**	Turkey	Positive	Diarrheal
**MB/BF 47**	Turkey	Positive	Diarrheal
**MB/BF 48**	Turkey	Positive	Diarrheal
**MB/BF 49**	Turkey	Positive	Diarrheal
**MB/BF 50**	Turkey	Positive	Diarrheal
**MB/BF 51**	Turkey	Positive	Diarrheal
**MB/BF 52**	Turkey	Positive	Diarrheal
**MB/BF 53**	Turkey	Positive	Diarrheal

**MB/BF 54**	Turkey	Positive	Diarrheal
**MB/BF 55**	Turkey	Positive	Diarrheal
**MB/BF 56**	Turkey	Positive	Diarrheal
**MB/BF 57**	Turkey	Positive	Diarrheal
**MB/N 1**	Turkey	Negative	Diarrheal
**MB/N 2**	Turkey	Negative	Diarrheal
**MB/N 3**	Turkey	Negative	Diarrheal
**MB/N 4**	Turkey	Negative	Diarrheal
**MB/N 5**	Turkey	Negative	Diarrheal
**MB/N 6**	Turkey	Negative	Diarrheal
**MB/N 7**	Turkey	Negative	Diarrheal
**MB/N 8**	Turkey	Negative	Diarrheal
**MB/N 9**	Turkey	Negative	Diarrheal
**MB/N 10**	Turkey	Negative	Diarrheal
**MB/N 11**	Turkey	Negative	Diarrheal
**MB/N 12**	Turkey	Negative	Diarrheal
**MB/N 13**	Turkey	Negative	Diarrheal
**MB/N 14**	Turkey	Negative	Diarrheal
**MB/N 15**	Turkey	Negative	Diarrheal
**MB/N 16**	Turkey	Negative	Diarrheal
**Korea 1**	South Korea	Negative	Diarrheal
**Korea 2**	South Korea	Negative	Diarrheal
**Korea 3**	South Korea	Negative	Diarrheal
**Korea 4**	South Korea	Negative	Diarrheal
**Korea 5**	South Korea	Negative	Diarrheal
**Korea 6**	South Korea	Negative	Diarrheal
**Korea 7**	South Korea	Negative	Diarrheal
**Korea 8**	South Korea	Negative	Diarrheal

**Korea 9**	South Korea	Negative	Diarrheal
**Korea 10**	South Korea	Negative	Diarrheal
**Korea 11**	South Korea	Negative	Diarrheal
**Korea 12**	South Korea	Negative	Diarrheal
**Korea 13**	South Korea	Negative	Diarrheal
**Korea 14**	South Korea	Negative	Diarrheal
**Korea 15**	South Korea	Negative	Diarrheal
**Korea 16**	South Korea	Negative	Diarrheal
**Korea 17**	South Korea	Negative	Diarrheal
**Korea 18**	South Korea	Negative	Diarrheal
**Korea 19**	South Korea	Negative	Diarrheal
**Korea 20**	South Korea	Negative	Diarrheal
**Korea 21**	South Korea	Positive	Non-diarrheal
**Korea 22**	South Korea	Positive	Non-diarrheal
**Korea 23**	South Korea	Positive	Non-diarrheal
**Korea 24**	South Korea	Positive	Non-diarrheal
**Korea 25**	South Korea	Positive	Non-diarrheal
**Korea 26**	South Korea	Positive	Non-diarrheal
**Korea 27**	South Korea	Positive	Non-diarrheal
**Korea 28**	South Korea	Positive	Non-diarrheal
**Korea 29**	South Korea	Positive	Non-diarrheal
**Korea 30**	South Korea	Positive	Non-diarrheal
**Korea 31**	South Korea	Positive	Non-diarrheal
**Korea 32**	South Korea	Positive	Non-diarrheal
**Korea 33**	South Korea	Positive	Non-diarrheal
**Korea 34**	South Korea	Positive	Non-diarrheal
**Korea 35**	South Korea	Positive	Non-diarrheal
**Korea 36**	South Korea	Positive	Non-diarrheal

**Korea 37**	South Korea	Positive	Non-diarrheal
**Korea 38**	South Korea	Positive	Non-diarrheal
**Korea 39**	South Korea	Positive	Non-diarrheal
**Korea 40**	South Korea	Positive	Non-diarrheal
**MFU2**	Thailand	Negative	Non-diarrheal
**MFU4**	Thailand	Negative	Non-diarrheal
**MFU7**	Thailand	Negative	Non-diarrheal
**MFU8**	Thailand	Negative	Non-diarrheal
**MFU10**	Thailand	Negative	Non-diarrheal
**MFU11**	Thailand	Negative	Non-diarrheal
**MFU12**	Thailand	Negative	Non-diarrheal
**MFU15**	Thailand	Negative	Non-diarrheal
**MFU16**	Thailand	Negative	Non-diarrheal
**MFU19**	Thailand	Negative	Non-diarrheal
**SNO_1**	Thailand	Negative	Non-diarrheal
**SNO_13**	Thailand	Negative	Non-diarrheal
**MFU3**	Thailand	Positive	Non-diarrheal
**MFU14**	Thailand	Positive	Non-diarrheal
**MFU17**	Thailand	Positive	Non-diarrheal
**SNO_4**	Thailand	Positive	Non-diarrheal
**SNO_6**	Thailand	Positive	Non-diarrheal
**SNO_17**	Thailand	Positive	Non-diarrheal
**SNO_22**	Thailand	Positive	Non-diarrheal
**SNO_23**	Thailand	Positive	Non-diarrheal
**SNO_24**	Thailand	Positive	Non-diarrheal
**SNO_26**	Thailand	Positive	Non-diarrheal
**SNO_27**	Thailand	Positive	Non-diarrheal
**SNO_28**	Thailand	Positive	Non-diarrheal

**SNO_29**	Thailand	Positive	Non-diarrheal
**SNO_33**	Thailand	Positive	Non-diarrheal
**MFU6**	Thailand	Negative	Non-diarrheal
**SNO_2**	Thailand	Negative	Non-diarrheal
**SNO_3**	Thailand	Negative	Non-diarrheal
**SNO_5**	Thailand	Negative	Non-diarrheal
**SNO_30**	Thailand	Negative	Non-diarrheal
**SNO_31**	Thailand	Negative	Non-diarrheal
**SNO_32**	Thailand	Negative	Non-diarrheal
**SNO_34**	Thailand	Negative	Non-diarrheal
**SNO_36**	Thailand	Negative	Non-diarrheal
**SNO_37**	Thailand	Negative	Non-diarrheal
**SNO_38**	Thailand	Negative	Non-diarrheal
**SNO_39**	Thailand	Negative	Non-diarrheal
**SNO_40**	Thailand	Negative	Non-diarrheal
**SNO_41**	Thailand	Negative	Non-diarrheal
**SNO_42**	Thailand	Negative	Non-diarrheal
**SNO_43**	Thailand	Negative	Non-diarrheal
**SNO_44**	Thailand	Negative	Non-diarrheal
**SNO_45**	Thailand	Negative	Non-diarrheal

Table S2. Significant metabolites identified across the three country cohorts. Those in green represent metabolites that were significantly increased in *B*+ samples as identified by univariate analysis. Those in blue were significantly decreased in the *B*+ samples when compared against *B*-. 5/30 metabolites had different expression results between at least two of the cohorts

Korea	Thailand	Turkey
	2-Hydroxyisovalerate	2-Hydroxyisovalerate
	2-Oxocaproate	2-Oxocaproate
	3-Methylglutarate	3-Methylglutarate
	3-Methylxanthine	3-Methylxanthine
	Acetamide	Acetamide
	Anthranilate	Anthranilate
	Arabinitol	Arabinitol
	Ascorbate	Ascorbate
Caprate		Caprate
	Creatinine	Creatinine
Dimethylamine	Dimethylamine	Dimethylamine
	Ethanolamine	Ethanolamine
	Galactarate	Galactarate
	Gentisate	Gentisate
	Glutamine	Glutamine
Glycerol	Glycerol	Glycerol
	Ibuprofen	Ibuprofen
	Isopropanol	Isopropanol
	Methylmalonate	Methylmalonate
	Na-Acetyllysine	Na-Acetyllysine
	Niacinamide	Niacinamide
	Oxypurinol	Oxypurinol
	Pipecolate	Pipecolate
	Pyroglutamate	Pyroglutamate
	Sebacate	Sebacate
	Suberate	Suberate

	Tiglylglycine	Tiglylglycine
	Trimethylamine N-oxide	Trimethylamine N-oxide
	Urea	Urea
Valine		Valine

Table S3. Listing all 98 significant metabolites identified from the *B+* vs *B-* sample cohort that were above the log2 fold change threshold. Univariate analysis was carried out with non-parametric tests on FDR-adjusted values (*p*-adj 0.05).

Metabolite	FC	log2(FC)	p.ajusted	-log10(p)
1,3-Dimethylurate	2.3432	1.2285	0.010317	1.9864
1,7-Dimethylxanthine	2.9837	1.5771	0.015624	1.8062
2-Aminobutyrate	12.325	3.6235	0.039807	1.4
2-Ethylacrylate	10.952	3.4531	0.00042761	3.3689
2-Hydroxyphenylacetate	2.0739	1.0523	0.016293	1.788
2-Oxobutyrate	0.45832	-1.1256	0.00018753	3.7269
2-Oxocaproate	0.13433	-2.8962	4.03E-05	4.3948
2-Phenylpropionate	0.095736	-3.3848	0.00012105	3.917
2'-Deoxyguanosine	3.4089	1.7693	0.015473	1.8104
2'-Deoxyuridine	2.1863	1.1285	0.0041995	2.3768
3-Hydroxybutyrate	0.20049	-2.3184	0.0028443	2.546
3-Hydroxykynurenine	0.31107	-1.6847	0.00059918	3.2224
3-Hydroxymandelate	2.2928	1.1971	0.0086029	2.0654
3-Indoxylsulfate	0.33435	-1.5806	0.00013963	3.855
3-Methylglutarate	2.7238	1.4456	0.0022112	2.6554
3-Methylxanthine	0.45392	-1.1395	0.00079219	3.1012
3,4-Dihydroxybenzeneacetate	3.9807	1.993	0.0012292	2.9104
3,4-Dihydroxymandelate	5.8713	2.5537	0.010422	1.9821
4-Hydroxy-3-methoxymandelate	3.1275	1.645	0.0099115	2.0039
5-Hydroxylysine	2.5873	1.3714	0.0032473	2.4885
Acetamide	2.0902	1.0636	0.00080579	3.0938
Acetoacetate	2.4306	1.2813	0.038043	1.4197
Agmatine	8.782	3.1346	0.0096886	2.0137
Alanine	4.7198	2.2387	0.0052514	2.2797
Alloisoleucine	2.691	1.4281	0.0038666	2.4127
Arginine	5.4029	2.4337	0.0060656	2.2171
Azelate	0.17921	-2.4803	4.03E-05	4.3945
beta-Alanine	22.655	4.5018	0.001153	2.9382
Cadaverine	2.0286	1.0205	0.00042761	3.3689
Caprate	0.10525	-3.2481	1.35E-08	7.8689
Caprylate	0.31112	-1.6845	0.00043142	3.3651
Carnitine	34.039	5.0891	0.011407	1.9428
Catechol	4.3152	2.1094	0.00016645	3.7787
Citraconate	6.5076	2.7021	0.00027344	3.5631
Citrate	3.0546	1.611	0.020785	1.6823
Desaminotyrosine	0.44287	-1.175	0.00011065	3.9561
Dimethyl sulfone	4.3721	2.1283	0.001153	2.9382

Dimethylamine	0.019985	-5.645	5.14E-06	5.2894
Ethanolamine	0.20544	-2.2832	5.38E-06	5.2691
Ethylene glycol	0.36233	-1.4646	0.001153	2.9382
Ethylmalonate	3.3644	1.7504	0.00058834	3.2304
Ferulate	2.0942	1.0664	0.025546	1.5927
Galactarate	4.7611	2.2513	0.027735	1.557
Galactonate	2.304	1.2041	0.024005	1.6197
Galactose	2.1337	1.0934	0.021956	1.6584
Gentisate	0.32007	-1.6435	4.72E-05	4.3259
Glucarate	4.4673	2.1594	0.0033493	2.475
Glucitol	11.071	3.4688	0.0032473	2.4885
Glycerol	0.16549	-2.5952	5.15E-08	7.2879
Glycine	8.8731	3.1494	0.018524	1.7323
Glycocholate	3.8387	1.9406	0.00013963	3.855
Glycolate	28.767	4.8463	0.045062	1.3462
Glycylproline	3.1928	1.6748	0.007361	2.1331
Guanidoacetate	8.5665	3.0987	0.016103	1.7931
Histamine	9.9222	3.3107	0.0022741	2.6432
Homocitrulline	3.0174	1.5933	0.0035856	2.4454
Isocaproate	0.41304	-1.2757	0.00018995	3.7214
Isoeugenol	3.0067	1.5882	0.0030342	2.518
Isoleucine	8.0192	3.0035	0.028444	1.546
Isopropanol	0.19718	-2.3424	1.12E-06	5.9503
Mandelate	38.966	5.2842	0.0025928	2.5862
Methanol	0.17077	-2.5499	8.30E-08	7.0808
Methylamine	2.1153	1.0809	0.00098811	3.0052
Methylmalonate	2.1728	1.1195	5.23E-05	4.2813
myo-Inositol	2.7509	1.4599	4.72E-05	4.3259
N-Acetyltyrosine	3.1057	1.6349	0.029146	1.5354
N-Carbamoylaspartate	9.2635	3.2116	0.048992	1.3099
N-Nitrosodimethylamine	0.43473	-1.2018	1.78E-06	5.7489
NADH	3.4074	1.7687	0.048907	1.3106
NADP+	2.9425	1.5571	0.0034568	2.4613
Nicotinamide N-oxide	2.1897	1.1308	4.24E-05	4.373
Nicotinurate	5.6684	2.5029	0.0091951	2.0364
O-Phosphoserine	2.0433	1.0309	5.25E-05	4.28
Ornithine	2.386	1.2546	0.00058834	3.2304
Pipecolate	0.097677	-3.3558	9.53E-06	5.0211
Proline	11.927	3.5761	0.0086625	2.0624
Quinolinate	2.142	1.0989	0.0015264	2.8163
Salicylurate	2.2051	1.1408	0.047783	1.3207

Sebacate	0.49188	-1.0236	1.29E-06	5.8899
Serine	16.977	4.0855	0.0028167	2.5503
Serotonin	12.507	3.6446	0.0058505	2.2328
sn-Glycero-3-phosphocholine	23.7	4.5668	0.0030342	2.518
Suberate	0.26649	-1.9078	4.71E-05	4.3265
Sucrose	0.39553	-1.3382	0.00016251	3.7891
Threonate	2.9945	1.5823	0.00011838	3.9267
Thymine	0.40958	-1.2878	0.00030514	3.5155
Thymol	0.42434	-1.2367	0.00023831	3.6229
trans-4-Hydroxy-L-proline	3.2879	1.7172	0.0054226	2.2658
trans-Aconitate	3.762	1.9115	0.022329	1.6511
Trimethylamine N-oxide	2.6035	1.3804	0.00016088	3.7935
Tropate	0.1741	-2.522	2.81E-05	4.5511
Tyramine	3.038	1.6031	0.00019293	3.7146
Urea	15.1	3.9165	0.0040541	2.3921
Valerate	0.47853	-1.0633	0.0059525	2.2253
Valine	10.873	3.4427	0.011699	1.9318
Valproate	0.23519	-2.0881	0.00013963	3.855
Vanillate	2.3265	1.2182	0.00026017	3.5847
Xylose	2.7722	1.471	4.72E-05	4.3259

Table S4. Listing all 166 significant metabolites identified from the D-*B*+ vs D+*B*- sample cohort that were above the log2 fold change threshold. Univariate analysis was carried out with non-parametric tests on FDR-adjusted values (*p*-adj 0.05).

Metabolite	FC	log2(FC)	p.ajusted	-log10(p)
Tryptophan	0.0064753	-7.2708	1.03E-05	4.9886
5-Aminopentanoate	0.087196	-3.5196	1.03E-05	4.9886
Malate	0.029474	-5.0844	5.20E-05	4.284
2-Aminobutyrate	0.09944	-3.33	5.24E-05	4.281
N-Carbamoyl-b-alanine	0.27032	-1.8872	5.24E-05	4.281
Valine	0.030959	-5.0135	5.68E-05	4.2457
Histamine	0.015501	-6.0114	7.10E-05	4.149
N-Carbamoylaspartate	0.40166	-1.3159	7.10E-05	4.149
O-Acetylcholine	0.016446	-5.9261	8.61E-05	4.0648
Acetoin	0.041546	-4.5891	8.61E-05	4.0648
4-Hydroxyphenylacetate	0.042539	-4.5551	8.61E-05	4.0648
Agmatine	0.12439	-3.0071	8.61E-05	4.0648
N-Acetylcysteine	0.13757	-2.8618	8.61E-05	4.0648
Ornithine	0.15953	-2.6481	8.61E-05	4.0648
Serine	0.20945	-2.2553	8.61E-05	4.0648
Acetone	0.029956	-5.061	0.00011526	3.9383
Isoleucine	0.065262	-3.9376	0.00011526	3.9383
Isocaproate	0.11237	-3.1537	0.00011526	3.9383
Cystathionine	0.29361	-1.768	0.00011526	3.9383
Glycolate	0.030184	-5.05	0.00014227	3.8469
Alanine	0.099347	-3.3314	0.00014227	3.8469
Glycylproline	0.13035	-2.9396	0.00014244	3.8464
5-Hydroxylysine	0.063254	-3.9827	0.00014402	3.8416
Pyroglutamate	0.15046	-2.7325	0.00015128	3.8202
1,6-Anhydro-b-D-glucose	0.014523	-6.1055	0.00015385	3.8129
Glucarate	0.089483	-3.4823	0.00015385	3.8129
Tyrosine	0.025489	-5.294	0.00016332	3.787
Syringate	0.085044	-3.5557	0.00016332	3.787
Ethylmalonate	0.42879	-1.2217	0.00016332	3.787
2-Aminoadipate	0.049745	-4.3293	0.00016356	3.7863
Catechol	0.054689	-4.1926	0.00016356	3.7863
2-Phosphoglycerate	0.33262	-1.588	0.00016356	3.7863
Urea	0.077289	-3.6936	0.00017856	3.7482
Glucose-1-phosphate	0.019494	-5.6808	0.00019351	3.7133

Pantothenate	0.096613	-3.3716	0.00019351	3.7133
Taurine	0.070452	-3.8272	0.00020884	3.6802
3-Hydroxyisobutyrate	0.038973	-4.6814	0.0002184	3.6608
Mandelate	0.069729	-3.8421	0.0002184	3.6608
Salicylate	0.049252	-4.3437	0.00022068	3.6562
Tyramine	0.09805	-3.3503	0.00022068	3.6562
Cadaverine	0.22688	-2.14	0.00022068	3.6562
Glycine	0.030692	-5.026	0.00022947	3.6393
Cholate	0.051877	-4.2688	0.00026676	3.5739
Anserine	0.11895	-3.0715	0.00032327	3.4904
Phenylalanine	0.02611	-5.2593	0.0003363	3.4733
beta-Alanine	0.025178	-5.3117	0.00034606	3.4608
Mannitol	0.035323	-4.8233	0.00034606	3.4608
Citrate	0.13153	-2.9266	0.00034606	3.4608
2-Methylglutarate	0.017032	-5.8756	0.0003506	3.4552
Alloisoleucine	0.13523	-2.8865	0.00036169	3.4417
myo-Inositol	0.016193	-5.9485	0.00037286	3.4285
2-Hydroxyisovalerate	0.099114	-3.3348	0.00037286	3.4285
2-Phenylpropionate	0.25871	-1.9506	0.00037286	3.4285
Propylene glycol	0.32711	-1.6121	0.00037286	3.4285
Arginine	0.22378	-2.1599	0.00037397	3.4272
Acetoacetate	0.063259	-3.9826	0.00037429	3.4268
Hydroxyacetone	0.14099	-2.8263	0.00040834	3.389
Aspartate	0.087638	-3.5123	0.00046565	3.3319
Carnosine	0.087193	-3.5197	0.00049303	3.3071
Melatonin	0.077057	-3.6979	0.0004975	3.3032
Serotonin	0.0067477	-7.2114	0.00049896	3.3019
Threonate	0.028682	-5.1237	0.000582	3.2351
4-Carboxyglutamate	0.091621	-3.4482	0.00060238	3.2201
Glutathione	0.079087	-3.6604	0.00062219	3.2061
Ethanolamine	0.12466	-3.0039	0.00062219	3.2061
3-Aminoisobutyrate	0.15519	-2.6879	0.00062219	3.2061
Dimethylamine	5.0052	2.3234	0.00062219	3.2061
Gentisate	0.36132	-1.4687	0.00062219	3.2061
Xanthine	0.081808	-3.6116	0.00071124	3.148
Gluconate	0.13922	-2.8446	0.00071852	3.1436
5-Methoxysalicylate	0.039778	-4.6519	0.00072025	3.1425
Sarcosine	0.20625	-2.2776	0.00092216	3.0352
1,3-Diaminopropane	0.038148	-4.7122	0.00099977	3.0001
Proline	0.031013	-5.011	0.0010984	2.9593
Methionine	0.022969	-5.4442	0.0012786	2.8932

Citraconate	0.015437	-6.0175	0.0013455	2.8711
Creatine phosphate	0.07838	-3.6734	0.0014	2.8539
Isocitrate	0.10656	-3.2302	0.0014778	2.8304
Methylmalonate	0.10842	-3.2053	0.0015561	2.808
Creatinine	0.020371	-5.6173	0.0016522	2.782
Tartrate	0.35473	-1.4952	0.0016522	2.782
5-Hydroxytryptophan	0.055367	-4.1748	0.0017746	2.7509
Saccharopine	0.18641	-2.4234	0.0017894	2.7473
Creatine	0.013553	-6.2053	0.001981	2.7031
pi-Methylhistidine	0.022082	-5.501	0.00213	2.6716
Quinolinate	0.089148	-3.4877	0.0021434	2.6689
O-Phosphoserine	0.072775	-3.7804	0.0022189	2.6539
trans-4-Hydroxy-L-proline	0.13246	-2.9164	0.0022945	2.6393
trans-Aconitate	0.16084	-2.6363	0.0023425	2.6303
5-Aminolevulinate	0.088668	-3.4954	0.0024461	2.6115
Lactate	0.082024	-3.6078	0.0025085	2.6006
2-Hydroxyisobutyrate	0.020254	-5.6256	0.0025226	2.5981
Biotin	0.074007	-3.7562	0.0025226	2.5981
2-Hydroxyglutarate	0.24805	-2.0113	0.0025226	2.5981
Histidine	0.041302	-4.5976	0.0025922	2.5863
2-Hydroxybutyrate	0.15813	-2.6609	0.0026943	2.5696
3-Methyl-2-oxovalerate	0.38452	-1.3789	0.0026943	2.5696
N-Acetylaspartate	0.11419	-3.1305	0.002712	2.5667
Homovanillate	0.12303	-3.0229	0.0027171	2.5659
Erythritol	0.18981	-2.3973	0.0027963	2.5534
Threonine	0.06448	-3.955	0.0032379	2.4897
Lactose	0.12275	-3.0262	0.0035807	2.446
2-Hydroxy-3-methylvalerate	0.30845	-1.6969	0.0038347	2.4163
Glutamate	0.08942	-3.4833	0.0040781	2.3895
Homocitrulline	0.19853	-2.3325	0.0041559	2.3813
Citrulline	0.14825	-2.7539	0.0044189	2.3547
Glucitol	0.011332	-6.4634	0.0047913	2.3196
Glutaric acid monomethyl ester	0.288	-1.7958	0.0050111	2.3001
4-Guanidinobutanoate	0.14631	-2.7729	0.005095	2.2929
Isobutyrate	0.15284	-2.7099	0.005095	2.2929
Ascorbate	0.12268	-3.027	0.0051948	2.2844
Isoeugenol	0.23945	-2.0622	0.0051948	2.2844
Xylitol	0.15274	-2.7109	0.0052433	2.2804
3,5-Dibromotyrosine	0.02308	-5.4372	0.0056153	2.2506

N6-Acetyllysine	0.20316	-2.2993	0.0056622	2.247
Glucose-6-phosphate	0.096187	-3.378	0.0065013	2.187
Galactarate	0.28395	-1.8163	0.006643	2.1776
Xanthurenate	0.11585	-3.1097	0.0067592	2.1701
Succinylacetone	0.17245	-2.5358	0.0067592	2.1701
3-Phenyllactate	0.12367	-3.0154	0.0072422	2.1401
sn-Glycero-3-phosphocholine	0.018608	-5.7479	0.0081437	2.0892
o-Cresol	0.075415	-3.729	0.0087883	2.0561
Fumarate	0.14244	-2.8116	0.0090016	2.0457
3-Methylglutarate	0.31829	-1.6516	0.009069	2.0424
O-Phosphoethanolamine	0.0089106	-6.8103	0.010815	1.966
4-Aminobutyrate	2.2609	1.1769	0.010815	1.966
Pyruvate	0.10514	-3.2496	0.012231	1.9125
Betaine	0.080019	-3.6435	0.012547	1.9015
Allantoin	0.1137	-3.1367	0.012647	1.898
4-Aminohippurate	0.186	-2.4266	0.013275	1.877
Trimethylamine	0.28129	-1.8299	0.013771	1.861
Galactonate	0.30246	-1.7252	0.013847	1.8587
Tiglylglycine	0.35794	-1.4822	0.01409	1.8511
Glycerate	0.05024	-4.315	0.014542	1.8374
3-Hydroxy-3-methylglutarate	0.099744	-3.3256	0.014542	1.8374
2-Ethylacrylate	0.21629	-2.209	0.014727	1.8319
Lactulose	0.11917	-3.0689	0.015406	1.8123
tau-Methylhistidine	0.062662	-3.9963	0.015661	1.8052
Urocanate	0.061245	-4.0293	0.015756	1.8026
Riboflavin	0.28728	-1.7995	0.015756	1.8026
Butanone	0.092802	-3.4297	0.01734	1.761
Trehalose	0.25472	-1.973	0.017735	1.7512
N-Acetyltyrosine	0.073225	-3.7715	0.018079	1.7428
Choline	0.095089	-3.3946	0.018198	1.74
Fructose	0.10321	-3.2763	0.018634	1.7297
NADPH	0.011286	-6.4693	0.020708	1.6839
O-Phosphocholine	0.11779	-3.0857	0.020708	1.6839
Imidazole	0.12154	-3.0405	0.020708	1.6839
5-Hydroxyindole-3-acetate	0.1763	-2.5039	0.024487	1.6111
Indole-3-lactate	0.21656	-2.2072	0.025433	1.5946
NADH	0.10792	-3.2119	0.025838	1.5877
N-Methylhydantoin	0.3113	-1.6836	0.027764	1.5565
5,6-Dihydrothymine	0.34175	-1.549	0.028379	1.547

Homocystine	0.49276	-1.0211	0.028637	1.5431
Arabinose	0.14465	-2.7893	0.031633	1.4999
2-Hydroxyvalerate	0.24483	-2.0301	0.033856	1.4704
3-Methylxanthine	0.31724	-1.6564	0.033856	1.4704
N-Isovaleroylglycine	0.14851	-2.7514	0.036257	1.4406
3-Hydroxykynurenine	3.61	1.852	0.036257	1.4406
1,7-Dimethylxanthine	0.10909	-3.1964	0.038387	1.4158
Caffeine	0.14031	-2.8333	0.038901	1.41
3,4-Dihydroxymandelate	0.17346	-2.5274	0.039551	1.4028
Acetylsalicylate	0.12166	-3.0391	0.040746	1.3899
NAD+	0.042684	-4.5501	0.044468	1.352
N-Acetylserotonin	0.38487	-1.3775	0.047499	1.3233
2-Hydroxyisocaproate	0.096102	-3.3793	0.048592	1.3134

Table S5. Listing all 6 significant metabolites identified from the D-*B*+ vs D-*B*- sample cohort that were above the log2 fold change threshold. Univariate analysis was carried out with non-parametric tests on FDR-adjusted values (*p*-adj 0.05).

Metabolite	FC	log2(FC)	p.ajusted	-log10(p)
Dimethylamine	7.268	2.8616	0.0011629	2.9345
Formate	2.6002	1.3786	0.0058908	2.2298
Glycerol	10.071	3.3322	0.0094025	2.0268
Niacinamide	4.1112	2.0396	0.010364	1.9845
Ethylene glycol	4.0168	2.006	0.012054	1.9189
Methanol	30.154	4.9143	0.023092	1.6365

Table S6. Listing all 51 significant metabolites identified from the B+(D+ and D-) excluding Korea D+ sample cohort that were above the log2 fold change threshold. Univariate analysis was carried out with non-parametric tests on FDR-adjusted values (*p*-adj 0.05).

Metabolite	FC	log2(FC)	p.ajusted	-log10(p)
Methanol	5.6077	2.4874	1.51E-05	4.8211
Dimethylamine	67.623	6.0794	2.95E-05	4.5307
Glycerol	5.6219	2.4911	2.95E-05	4.5307
Caprate	6.7302	2.7506	5.62E-05	4.2507
Thymine	8.3579	3.0631	0.00034693	3.4598
Azelate	10.725	3.4229	0.0014216	2.8472
Ibuprofen	4.1123	2.0399	0.0014216	2.8472
Isopropanol	4.5595	2.1889	0.0018016	2.7443
Xylose	0.33653	-1.5712	0.0030442	2.5165
Ethanolamine	3.5951	1.846	0.003249	2.4883
Methylguanidine	2.8991	1.5356	0.0058903	2.2299
Pipecolate	9.4372	3.2384	0.0068859	2.162
Glycocholate	0.23462	-2.0916	0.0071023	2.1486
4-Hydroxy-3-methoxymandelate	0.3773	-1.4062	0.0071023	2.1486
2-Hydroxyisobutyrate	5.1889	2.3754	0.0076169	2.1182
myo-Inositol	0.28738	-1.799	0.0089813	2.0467
Vanillate	0.41785	-1.2589	0.0099369	2.0028
Tropate	5.2946	2.4045	0.011018	1.9579
Methylmalonate	0.48117	-1.0554	0.011047	1.9568
Histamine	0.082246	-3.6039	0.012418	1.906
3-Methylxanthine	2.3428	1.2282	0.012418	1.906
Trimethylamine N-oxide	0.36614	-1.4495	0.012422	1.9058
Ethylene glycol	2.9668	1.5689	0.013608	1.8662
Suberate	2.7252	1.4464	0.014021	1.8532
2-Oxocaproate	6.4484	2.6889	0.014238	1.8466
Urea	3.382	1.7579	0.01617	1.7913
3-Indoxylsulfate	3.0308	1.5997	0.016179	1.7911
Gentisate	2.4963	1.3198	0.016179	1.7911
Glycerate	0.45804	-1.1265	0.016371	1.7859
2-Phenylpropionate	10.331	3.3689	0.0182	1.7399
Oxypurinol	2.3398	1.2264	0.019816	1.703
O-Phosphoserine	0.35184	-1.507	0.020555	1.6871
Benzoate	0.24348	-2.0381	0.021948	1.6586
3-Methylglutarate	3.5607	1.8322	0.021965	1.6583

Creatinine	5.0101	2.3248	0.02214	1.6548
N-Acetylglutamine	0.41526	-1.2679	0.02214	1.6548
Pyroglutamate	2.1607	1.1115	0.023205	1.6344
Hydroxyacetone	0.44135	-1.18	0.026973	1.5691
Caprylate	6.612	2.7251	0.027409	1.5621
N-Acetylserotonin	0.4305	-1.2159	0.029847	1.5251
Glutaric acid monomethyl ester	2.1003	1.0706	0.030869	1.5105
Threonate	0.23542	-2.0867	0.033517	1.4747
Thymol	2.0768	1.0544	0.033517	1.4747
Citraconate	0.11474	-3.1236	0.033889	1.4699
Sucrose	3.7947	1.924	0.033889	1.4699
Protocatechuate	0.45972	-1.1212	0.033889	1.4699
Valproate	3.8092	1.9295	0.03545	1.4504
Lactate	0.39322	-1.3466	0.037848	1.422
Catechol	0.16268	-2.6199	0.041385	1.3832
3-Hydroxykynurenine	3.0838	1.6247	0.041722	1.3796
trans-4-Hydroxy-L-proline	0.36018	-1.4732	0.045623	1.3408

Table S7. Details of pathway analysis for the Thai cohort, only values with p-adjust <0.05 are included. The 'Total' column equates to the total number of compounds in the given pathway and the 'Hits' are compounds from this dataset that match to a given pathway. The p-adjust values are adjusted Raw p-values following the Holm-Bonferroni method. FDR is false discovery rate adjusted p-value and the Impact is the impact value was calculated from pathway topology analysis.

Pathway	Total Cmpd	Hits	Raw p	-LOG10(p)	Holm adjust	FDR	Impact
Pentose and glucuronate interconversions	18	7	9.18E-06	5.037	0.00049594	0.00049594	0.57812
Galactose metabolism	27	9	2.69E-05	4.57	0.0014264	0.00072668	0.13634
Amino sugar and nucleotide sugar metabolism	37	9	8.14E-05	4.0892	0.0042347	0.0014658	0.28103
Tyrosine metabolism	42	11	0.00034722	3.4594	0.017708	0.0046875	0.24403
Ascorbate and aldarate metabolism	8	5	0.00047618	3.3222	0.023809	0.0051427	1
Starch and sucrose metabolism	18	5	0.00083072	3.0805	0.040705	0.0072703	0.33334
Phosphatidylinositol signaling system	28	1	0.00099831	3.0007	0.047919	0.0072703	0.03736

Table S8. Details of pathway analysis for the Turkey cohort, only values with *p*-adjust <0.05 are included. The 'Total' column equates to the total number of compounds in the given pathway and the 'Hits' are compounds from this dataset that match to a given pathway. The *p*-adjust values are adjusted Raw *p*-values following the Holm-Bonferroni method. FDR is false discovery rate adjusted *p*-value and the Impact is the impact value was calculated from pathway topology analysis

Pathway	Total Cmpd	Hits	Raw p	-LOG10(p)	Holm adjust	FDR	Impact
Aminoacyl-tRNA biosynthesis	48	19	0.00000	15.878	0.00000	0.00000	0.00000
Alanine, aspartate and glutamate metabolism	28	11	0.00000	8.1308	0.00000	0.00000	0.62340
Glycine, serine and threonine metabolism	33	15	0.00000	6.8597	0.00001	0.00000	0.55748
Arginine and proline metabolism	38	11	0.00000	6.7482	0.00001	0.00000	0.44293
Selenocompound metabolism	20	1	0.00000	6.6831	0.00001	0.00000	0.00000
Pantothenate and CoA biosynthesis	19	7	0.00000	6.4734	0.00002	0.00000	0.05714
Arginine biosynthesis	14	10	0.00000	6.3463	0.00002	0.00000	0.48223
Lysine degradation	25	5	0.00000	6.3226	0.00002	0.00000	0.18780
Histidine metabolism	16	8	0.00000	6.1662	0.00003	0.00000	0.67212
Primary bile acid biosynthesis	46	4	0.00000	5.8995	0.00006	0.00001	0.02321
Glyoxylate and dicarboxylate metabolism	32	12	0.00000	5.6572	0.00010	0.00001	0.32012
Glutathione metabolism	28	8	0.00000	5.4103	0.00017	0.00002	0.40365
Valine, leucine and isoleucine degradation	40	8	0.00000	5.3959	0.00017	0.00002	0.07143
Pyrimidine metabolism	39	13	0.00000	5.3916	0.00017	0.00002	0.37937
Valine, leucine and isoleucine biosynthesis	8	7	0.00001	5.0678	0.00035	0.00003	0.00000
beta-Alanine metabolism	21	8	0.00001	5.0623	0.00035	0.00003	0.55970

Porphyrin and chlorophyll metabolism	30	3	0.00001	4.8985	0.00049	0.00004	0.02799
D-Glutamine and D-glutamate metabolism	6	2	0.00002	4.7804	0.00063	0.00005	0.50000
Nitrogen metabolism	6	2	0.00002	4.7804	0.00063	0.00005	0.00000
Tyrosine metabolism	42	11	0.00005	4.309	0.00177	0.00013	0.20399
Biotin metabolism	10	2	0.00012	3.9162	0.00424	0.00032	0.20000
Cysteine and methionine metabolism	33	9	0.00017	3.7748	0.00571	0.00042	0.50713
Propanoate metabolism	23	5	0.00035	3.4516	0.01167	0.00085	0.04061
Thiamine metabolism	7	1	0.00047	3.329	0.01500	0.00107	0.00000
Tryptophan metabolism	41	9	0.00053	3.278	0.01635	0.00116	0.55537
Phenylalanine metabolism	10	5	0.00082	3.0854	0.02465	0.00174	0.35714

Table S9. Details of pathway analysis for the South Korean cohort, only values with p-adjust <0.05 are included. The 'Total' column equates to the total number of compounds in the given pathway and the 'Hits' are compounds from this dataset that match to a given pathway. The p-adjust values are adjusted Raw p-values following the Holm-Bonferroni method. FDR is false discovery rate adjusted p-value and the Impact is the impact value was calculated from pathway topology analysis

Pathway	Total Cmpd	Hits	Raw p	-LOG10(p)	Holm adjust	FDR	Impact
Glyoxylate and dicarboxylate metabolism	32	13	0.00035	3.4547	0.01930	0.00764	0.36245
Pyruvate metabolism	22	5	0.00040	3.3963	0.02168	0.00764	0.29859
Histidine metabolism	16	8	0.00053	3.2725	0.02830	0.00764	0.67212
Glycerolipid metabolism	16	3	0.00056	3.2554	0.02888	0.00764	0.33022

Table S10. Details of pathway analysis for the *B*+ vs *B*- samples, only values with p-adjust <0.05 are included. The 'Total' column equates to the total number of compounds in the given pathway and the 'Hits' are compounds from this dataset that match to a given pathway. The p-adjust values are adjusted Raw p-values following the Holm-Bonferroni method. FDR is false discovery rate adjusted p-value and the Impact is the impact value was calculated from pathway topology analysis

Pathway	Total Cmpd	Hits	Raw p	-LOG10(p)	Holm adjust	FDR	Impact
Aminoacyl-tRNA biosynthesis	48	19	9.57E-07	6.0191	5.26E-05	5.26E-05	0
Glycine, serine and threonine metabolism	33	15	8.11E-06	5.0909	0.00043806	0.00020308	0.55748
Phenylalanine metabolism	10	5	1.42E-05	4.8476	0.00075275	0.00020308	0.35714
Histidine metabolism	16	8	1.48E-05	4.8306	0.00076803	0.00020308	0.67212
Fatty acid biosynthesis	47	3	1.87E-05	4.7271	0.00095612	0.00020622	0
Primary bile acid biosynthesis	46	4	6.22E-05	4.206	0.0031118	0.00053344	0.02321
Selenocompound metabolism	20	1	6.79E-05	4.1682	0.0033267	0.00053344	0
Porphyrin and chlorophyll metabolism	30	3	0.00015148	3.8197	0.0072708	0.00079889	0.02799
Glycerolipid metabolism	16	3	0.00015255	3.8167	0.0072708	0.00079889	0.33022
Valine, leucine and isoleucine biosynthesis	8	7	0.00015955	3.7972	0.007337	0.00079889	0
beta-Alanine metabolism	21	9	0.00017659	3.753	0.0079465	0.00079889	0.61567
Glyoxylate and dicarboxylate metabolism	32	12	0.00017748	3.7508	0.0079465	0.00079889	0.32012

Phenylalanine, tyrosine and tryptophan biosynthesis	4	2	0.00019207	3.7165	0.008259	0.00079889	1
Arginine and proline metabolism	38	12	0.00020335	3.6917	0.0085409	0.00079889	0.46678
Tyrosine metabolism	42	12	0.00024276	3.6148	0.0099532	0.00085455	0.26866
Pantothenate and CoA biosynthesis	19	8	0.0002486	3.6045	0.0099532	0.00085455	0.07857
Ubiquinone and other terpenoid-quinone biosynthesis	9	2	0.00036397	3.4389	0.014195	0.0011776	0
Glycerophospholipid metabolism	36	6	0.00064487	3.1905	0.024505	0.0019704	0.1208
Purine metabolism	65	17	0.00074246	3.1293	0.027471	0.0021492	0.28907
Alanine, aspartate and glutamate metabolism	28	13	0.0011508	2.939	0.04143	0.0031648	0.75802
Nicotinate and nicotinamide metabolism	15	8	0.0012919	2.8888	0.045217	0.0033836	0.77764
Galactose metabolism	27	9	0.0014234	2.8467	0.048394	0.0035584	0.13634
